# Common but Nonpersistent Acquisitions of Plant Viruses by Plant-Associated Fungi

**DOI:** 10.3390/v14102279

**Published:** 2022-10-17

**Authors:** Xinran Cao, Jie Liu, Jianguo Pang, Hideki Kondo, Shengqi Chi, Jianfeng Zhang, Liying Sun, Ida Bagus Andika

**Affiliations:** 1 College of Plant Health and Medicine, Qingdao Agricultural University, Qingdao 266109, China; 2 University Library, Northwest A&F University, Xianyang 712100, China; 3 Institute of Plant Science and Resources, Okayama University, Kurashiki 710-0046, Japan; 4 State Key Laboratory of Crop Stress Biology for Arid Areas and College of Plant Protection, Northwest A&F University, Xianyang 712100, China

**Keywords:** plant viruses, fungi, virus acquisition, cross-infection, virus transmission

## Abstract

Investigating a virus’s host range and cross-infection is important for better understanding the epidemiology and emergence of viruses. Previously, our research group discovered a natural infection of a plant RNA virus, cumber mosaic virus (genus *Cucumovirus*, family *Bromoviridae*), in a plant pathogenic basidiomycetous fungus, *Rhizoctonia solani*, isolated from a potato plant grown in the field. Here, we further extended the study to investigate whether similar cross-infection of plant viruses occurs widely in plant-associated fungi in natural conditions. Various vegetable plants such as spinach, leaf mustard, radish, celery, and other vegetables that showed typical virus-like diseases were collected from the fields in Shandong Province, China. High-throughput sequencing revealed that at least 11 known RNA viruses belonging to different genera, including *Potyvirus*, *Fabavirus*, *Polerovirus*, *Waikavirus*, and *Cucumovirus*, along with novel virus candidates belonging to other virus genera, infected or associated with the collected vegetable plants, and most of the leaf samples contained multiple plant viruses. A large number of filamentous fungal strains were isolated from the vegetable leaf samples and subjected to screening for the presence of plant viruses. RT-PCR and Sanger sequencing of the PCR products revealed that among the 169 fungal strains tested, around 50% were carrying plant viruses, and many of the strains harbored multiple plant viruses. The plant viruses detected in the fungal isolates were diverse (10 virus species) and not limited to particular virus genera. However, after prolonged maintenance of the fungal culture in the laboratory, many of the fungal strains have lost the virus. Sequencing of the fungal DNA indicated that most of the fungal strains harboring plant viruses were related to plant pathogenic and/or endophytic fungi belonging to the genera *Alternaria*, *Lecanicillium,* and *Sarocladium*. These observations suggest that the nonpersistent acquisition of plant viruses by fungi may commonly occur in nature. Our findings highlight a possible role for fungi in the life cycle, spread, and evolution of plant viruses.

## 1. Introduction

Plant viruses cause major losses in agricultural production worldwide by reducing crop yields and/or the quality of agricultural products [1]. Nevertheless, aside from utilizing genetic sources of plant resistance or tolerance against viruses [2,3,4], there has been no breakthrough in the invention of effective control methods for crop viral diseases, particularly through chemical treatment. Therefore, detailed investigations of epidemiological characteristics such as the distribution, host range, host jumps, reservoirs, and transmission pathways of plant viruses in nature can lead to a better understanding of plant virus emergence and serve as a basis for the development of alternative control measures and management of plant viral diseases in the agricultural setting [5,6,7,8].

Generally, plant viruses are thought to exist in and infect only plant species, including cultivated and wild plants, although there are a few exceptions wherein viruses such as tospo-, tenui-, rhabdo-, and reoviruses belonging to negative-sense singe-stranded (ss) or double-stranded (ds) RNA viral genera infect and multiply in their insect vectors [9,10]. However, accumulating findings regarding phylogenetic relationships between plant and fungal viruses, artificial inoculations, and the detection of natural infections of plant viruses support the view that the host range of plant viruses may extend to the fungal species [11]. For example, fungal viruses that highly resemble plant potexviruses (genus *Potexvirus,* family *Alphaflexiviridae*) have been isolated from ascomycete fungi, *Botrytis cinerea,* and *Sclerotinia sclerotiorum* [12,13,14], suggesting the recent transmission of plant viruses to fungi. Indeed, artificial inoculations showed that fungi are suitable hosts of plant viruses of the phylum *Kitrinoviricota*, such as in the case of the replication of brome mosaic virus (genus *Bromovirus*, family *Bromoviridae*) and tomato bushy stunt virus (genus *Tombusvirus*, family *Tombusviridae*) in *Saccharomyces cerevisiae* (yeast), a unicellular fungus [15,16], and the replication of tobacco mosaic virus (TMV, genus *Tobamovirus*, family *Virgaviridae*) and other plant RNA viruses in plant pathogenic filamentous ascomycetes fungi, *Colletotrichum acutatum,* and *Fusarium graminearum* as well as in the plant pathogenic oomycete *Phytophthora infestans* [17,18,19]. Moreover, by artificial inoculation, some viroids, subviral agents of plants, were shown to infect plant pathogenic ascomycetes, *Cryphonectria parasitica*, *Valsa mali,* and *F. graminearum,* as well as *P. infestans* [20,21]. Importantly, our research group discovered the natural infection of a plant virus in a plant pathogenic basidiomycetous fungus. One of the *Rhizoctonia solani* strains isolated from potato plants grown in the field was found to be infected with a plant virus, cucumber mosaic virus (CMV, genus *Cucumovirus,* family *Bromoviridae*) [22]. This finding represents the first evidence of naturally occurring cross-infection of a plant virus in a fungus.

Under laboratory conditions, CMV, TMV, viroid, and the fungal virus Cryphonectria hypovirus 1 (genus *Hypovirus*, family *Hypoviridae*) can be bidirectionally transferred between the plant and fungus or oomycete during the fungal colonialization of plants [19,20,21,22]. This observation is in line with the current knowledge that during fungal infection, plants and fungi interact by exchanging cellular contents, including various macromolecules such as protein effectors and small RNAs [23,24]. In natural ecosystems, land plants, especially those in agricultural conditions, are commonly colonialized by a wide variety of fungi, including phytopathogens, mycorrhizas, and endophytes [25,26,27,28]. Thus, the probabilities of coinfection of an individual plant with fungi and plant viruses that leads to the cross-infection of plant viruses in fungi in nature are considerable.

In light of our first finding of the natural cross-infection of a plant virus in a fungus, the question arises as to how common occurrences of cross-infection of plant viruses in fungi are in nature. In this study, we carried out a systematic screening for fungal strains harboring plant viruses. A large number of fungal strains were isolated from the leaves of various vegetable plants with viral infections, and the presence of plant viruses in the fungal strains was examined. The results showed that about half of the isolated fungal strains were carrying plant viruses, and the plant viruses detected in the fungal isolates were diverse and belonged to various virus genera. Our results revealed the common acquisition of plant viruses by plant-associated fungi in nature. The relevance of the cross-infection of plant viruses in fungi in the context of the reservoir and transmission of plant viruses in nature is further discussed.

## 2. Materials and Methods

### 2.1. Plant Samples

Leaf mustard (*Brassica juncea*, Brassicales), napa cabbage (*B. rapa* var. *glabra*, Brassicales), bok choy (*B. rapa* var. *chinensis*, Brassicales), radish (*Raphanus sativus* var. *longipinnatus*, Brassicales), stem lettuce (*Lactuca sativa* var. *augustana,* Asterales), celery (*Apium graveolens,* Apiales), spinach (*Spinacia oleracea*, Caryophyllales), and watermelon (*Citrullus lanatus*, Cucurbitales) plants showing typical virus-like disease symptoms were collected from several neighboring fields located in Zhangjiashagou Village, Chengyang District, Qingdao City, Shandong Province, China (36°27′ N and 120°48′ E) in November 2019.

### 2.2. RNA and Total Nucleic Acid Extraction

Plant total RNA was extracted from leaf tissues using Trizol (Invitrogen, Waltham, MA, USA) according to the manufacturer’s protocols. The total nucleic acid of fungi was extracted from mycelia cultured in potato dextrose agar (PDA, Becton, Dickinson & Co., Mountain View, CA, USA) with cellophane for 3–5 days using the phenol-based method described previously [29]. 

### 2.3. Isolation of Fungal Strains from Leaves and Fungal Culture

Leaves were cut into small squares (0.5–0.8 cm) and successively washed with sterilized water and 75% alcohol before submersion in fresh 75% alcohol for 1 min. The leaves were rinsed with water, then submerged in 2% sodium hypochlorite for 2 min or 0.1% mercury (II) chloride for 5 min. The leaves were then rinsed with fresh 75% alcohol (once) and water (three times), dried with paper towels, placed on PDA plates, and left on the benchtop at room temperature. Once the fungi had grown, the fungal strains were subcultured by placing small mycelial plugs derived from the edge of the fungal colony on fresh PDA plates. Fungal strains were further maintained in the laboratory by subculturing similarly, as described previously [22]. 

### 2.4. High-Throughput Sequencing and Bioinformatic Analysis

For the preparation of the cDNA library, 2 μg of qualified total RNA was digested with 5U DNase I (Takara, Dalian, China) at 37 °C for 30 min then purified with RNeasy MinElute Cleanup Kit (Qiagen, Hilden, Germany) and eluted with 11 µL RNase-free water. The removal of ribosomal RNA was performed using a Ribo-off rRNA Depletion Kit (plant) (Vazyme, Nanjing, China) as follows: hybridization of RNA with a probe by adding rRNA Probe (Plant) and probe buffer under hybridization conditions at 95 °C for 2 min, then steadily decreasing from 95 °C to 22 °C (0.1 °C per second), and finally 5 min at 22 °C. RNA was then treated with RNase H at 37 °C for 30 min to specifically hydrolyze the RNA in the DNA–RNA duplexes, and DNase I at 37 °C for 30 min. The ribosomal-deleted RNA was purified using VAHTS RNA Clean Beads (Vazyme, Nanjing, China). The rRNA-depleted RNA (100 ng) was used for cDNA library construction using NEBNext Ultra Directional RNA Library Prep Kit for Illumina (NEB, Ipswich, MA, USA) according to the manufacturer’s protocol. After the construction of the library, three tests were carried out to ensure the quality of the library: Qubit fluorometer (Thermo Fisher Scientific Inc, Cleveland, OH, USA) quantification, 2% agarose gel electrophoresis detection, and high-sensitive DNA chip detection. The cDNA library (10 ng) was used for cluster generation carried out on a cBot Cluster Generation System using TruSeq PE Cluster Kit (Illumina, San Diego, CA, USA), and then bidirectional sequencing was carried out on the Illumina Hiseq/Miseq systems. RNA sequencing (RNA-seq) was carried out by Sangon Biotech Co., Ltd. (Shanghai, China). 

The Trinity software [30] was used for the de novo assembly of sequence reads. The assembled contigs were used as queries for BLASTX (local BLAST) searches against virus genome sequences (reference proteins) in the NCBI database. HISAT2 [31] was used to map reads to the assembled sequences. Samtools [32] was used to obtain data on mapped reads in order to determine the abundance of each contig.

### 2.5. Phylogenetic Analysis

Viral RNA dependent RNA Polymerase RdRP or L protein amino acid sequences were subjected to the maximum-likelihood (ML) tree construction. Multiple amino acid sequence alignments were generated by MAFFT version 7 (https://mafft.cbrc.jp/alignment/server/, accessed on 26 August 2022) [33]. Trimming of poorly reliable regions in the alignments was carried out using trimAl version 1.3 (http://phylemon.bioinfo.cipf.es, accessed on 26 August 2022) [34]. Alignments were then used to generate ML trees using PhyML 3.0 (http://www.atgc-montpellier.fr/phyml/, accessed on 26 August 2022) [35] with a best-fit model selected by the Smart Model Selection [36]. Finally, the ML trees were visualized and refined in FigTree v1.4.4 (http://tree.bio.ed.ac.uk/software/figtree/, accessed on 26 August 2022).

### 2.6. RT-PCR and Northern Blot Analyses

All primers used for reverse transcription (RT) and polymerase chain reaction (PCR) amplification in this study are listed in Supplementary Table S1. cDNA synthesis (20 µL) was performed using 0.5–1 µg total fungal nucleic acid and the reverse transcriptase M-MLV (Takara, Otsu, Japan). PCR amplification (20 µL) was performed using 2 µL cDNA solution as a template and 2× Accurate Taq Master Mix Dye Plus (Accurate Biotechnology, Hunan, China). The PCR conditions were as follows: 94 °C for 3 min; 34 cycles at 94 °C for 30 s, 52 °C for 30 s, and 72 °C for 60 s; and 72 °C for 10 min. For the secondary nested PCR amplification (20 µL), 2 µL product of the first PCR and 2× Accurate Taq Master Mix Dye Plus were used, and the conditions were as follows: 94 °C for 3 min; 34 cycles at 94 °C for 30 s, 52 °C for 30 s, and 72 °C for 30 s; and 72 °C for 10 min. The PCR products were then subjected to Sanger dideoxy sequencing.

For Northern blot analysis, total RNA (~10 µg) was denatured and subjected to denaturing agarose gel (1%) electrophoresis. RNA was transferred to a nitrocellulose membrane and viral RNA was detected using a random ^32^P-labeled CMV-specific probe (conserved 234–238 nt 3′-terminal regions of RNA1, RNA2, and RNA3 amplified from the CMV isolate obtained from this study) prepared using the Prime-a-Gene Labeling System (Promega, Madison, WI, USA) according to the manufacturer’s protocol. The radioactive bands were visualized with a Storm 820 Phosphorimager (GE Healthcare, Chicago, IL, USA).

### 2.7. Sequencing of Fungal DNA

For fungal species identification, the intergenic spacer (ITS) region of the nuclear ribosomal RNA genes was PCR-amplified using ITS1 and ITS4 primers [37], and translational elongation factor 1α (TEF1α) was PCR-amplified using EF1-1018F and EF1-1620R primers [38]. The PCR conditions were as follows: 94 °C for 3 min; 34 cycles at 94 °C for 30 s, 54 °C for 30 s, and 72 °C for 60 s; and 72 °C for 10 min. The PCR products were subjected to Sanger dideoxy sequencing and the obtained sequences were used as queries for BLASTn searches against GenBank standard databases (nt) or fungal ITS databases from fungi type and reference materials.

## 3. Results

### 3.1. Experimental Procedures and Collection of Plant Samples

**Figure 1:**
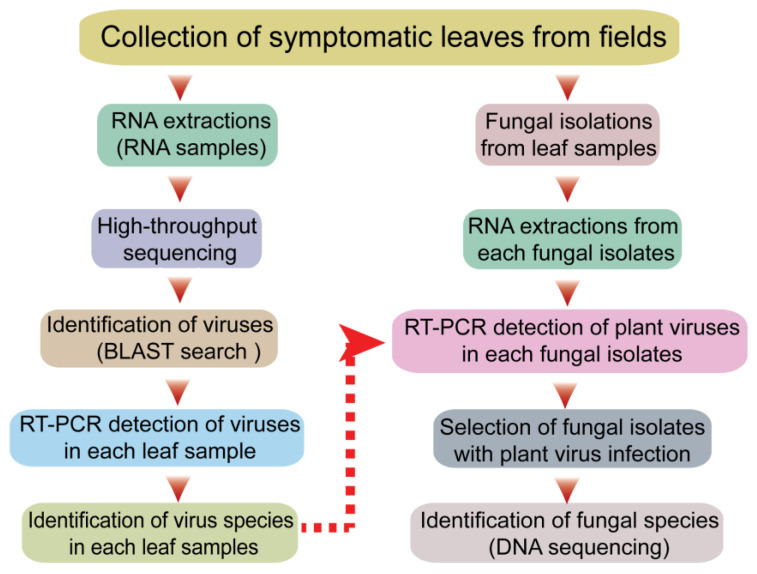
Schematic diagram illustrating the experimental procedures for the screening of fungal strains with plant virus infections.

**Figure 2:**
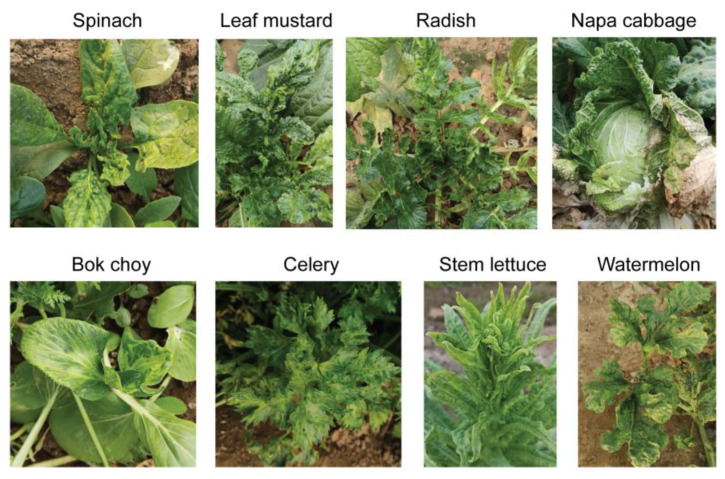
Representative vegetable plants showing typical virus-like disease symptoms collected as the sample material.

The sequential experimental procedures carried out in this study are illustrated in the schematic diagram shown in Figure 1. First, plants with typical virus-like disease symptoms were collected from the fields in Qingdao City, China. The leaf sample materials included various vegetable plants such as spinach, leaf mustard, radish, celery, napa cabbage, bok choy, stem lettuce, and watermelon showing mosaic, yellowing or chlorosis, leaf curly, vein clearing on leaves, and/or plant stunting (Figure 2). Each leaf sample consisted of pooled leaves collected from multiple plants belonging to the same species showing similar symptoms of viral infection. The leaf samples of some plants such as leaf mustard and radish were differently grouped based on their symptoms (numbered for each sample). It was noted that these vegetable plants did not show any discernable fungal-like disease symptoms or insect infestations (data not shown).

Total RNA was then extracted from a representative portion of all leaf samples (collected from 11 plant species), and another portion of the same leaf samples was used for the isolation of fungal strains from the leaf tissue (Figure 1). The total RNA extracted from different leaf samples (11 samples) was combined and then subjected to RNA-seq analysis. The obtained sequence contigs were used as queries for BLAST searches against virus databases. Based on the information on virus sequences derived from the BLAST searches (see below), RT-PCR assays were carried out to identify the viruses that infected or were associated with each leaf sample. In the other part of the work, after fungal isolation and subsequent subculturing, total RNA was extracted from the fungal strains and used for the RT-PCR-based detection of the plant viruses that were identified in the leaf sample from which the fungus was isolated. If fungal strains harboring plant viruses were identified, the fungal species was determined by DNA sequencing. 

### 3.2. Identification of Virus Species Present in Plant Samples

BLAST searches of the assembled sequence contigs (293,736 total contigs) derived from the RNA-seq data set (37,556,635 total reads) revealed numerous virus-related sequences (Supplementary File S1). As listed in Table 1, among the sequences, 11 belonged to known positive-sense ssRNA plant viruses, including five potyviruses (zucchini yellow mosaic virus [ZYMV], papaya ringspot virus [PRSV], turnip mosaic virus [TuMV], konjac mosaic virus [KoMV], and watermelon mosaic virus [WMV]) belonging to the family *Potyviridae*, one polerovirus (brassica yellows virus [BrYV]) belonging to the family *Solemoviridae* and a polerovirus-associated subviral RNA (turnip yellows virus-associated RNA [TuYV-aRNA]), one fabavirus (broad bean wilt virus 2 [BBWV-2], family *Secoviridae*), one waikavirus (brassica napus RNA virus 1 [BnRV1], family *Secoviridae*), one umbravirus (ixeridium yellow mottle virus 2 [IxYMV-2], family *Tombusviridae*), and CMV. Selected virus-related sequence contigs have been deposited to the GenBank/EMBL/DDBJ databases (Table 1). These plant virus sequences show significant nucleotide/amino acid sequence identities (78.4/88.1%~99.5/99.5% nt/aa sequence identities and data not shown) to virus isolates deposited in the GenBank database (Table 1 and data not shown). Among these known plant viruses, CMV was predominant in the pooled sample (87% of total virus reads; two subgroups were included), followed by BBWV2 (4.3%) and potyviruses (6.9%; Supplementary Figure S1). Besides the abovementioned viruses, other plant viruses, such as gammacarmoviruses, partitiviruses, and a varicosavirus, were also associated with the leaf samples, possibly as minor populations (Supplementary File S1 and data not shown). 

**Figure 3:**
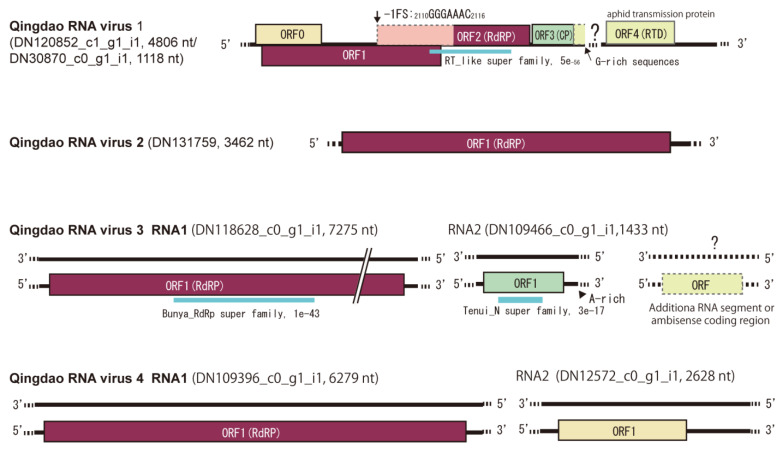
Schematic genome structures of four novel RNA viruses (named Qingdao RNA virus 1–4, QRV1–4) detected in the leaf samples. The name and length of sequence contigs from which the virus genomes were built are presented. A putative missing region (or segment) of ORV1 and possible RNA segments of QRV3 and ORV4 are also presented. The bold line and dashed line represent the genome sequence and unknown sequences of the 5′ and 3′ terminal regions. The colored boxes represent predicted open reading frames (ORFs). The conserved domains in the predicted viral proteins are shown with a blue bar along with the domain name and its *E-value* according to the NCBI conserved domain database (https://www.ncbi.nlm.nih.gov/Structure/cdd/wrpsb.cgi, accessed on 26 August 2022).

**Figure 4:**
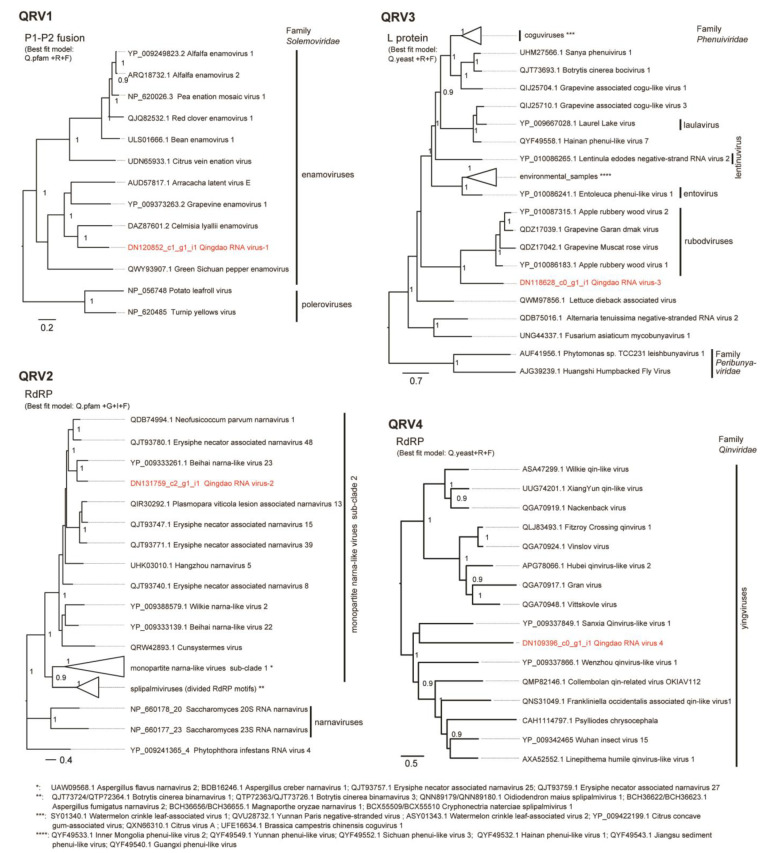
Phylogenetic relationships of QRV1, QRV2, QRV3, and QRV4 with their related viruses. The virus names are followed by their accession numbers. Poleroviruses, narnaviruses, and peribunyaviruses were used as outgroups in the tree for QRV1, QRV2, and QRV3, respectively. The trees were refined using FigTree ver. 1.3.1 and the scale bar represents amino acid distances. The numbers at the nodes indicate aLRT values determined using an SH-like calculation (>0.9 are displayed). Some phylogroups were collapsed into a triangle. The names and accession numbers of these viruses are shown below the trees.

Four virus-related sequence contigs had low or moderate amino acid sequence identities (22–64%) to known viruses and, therefore, were provisionally identified as Qingdao RNA virus 1–4 (QRV1, QRV2, QRV3, and QRV4; Table 1). QRV1-encoded putative RNA-dependent RNA polymerase (RdRP) had a 67.3% identity to that encoded by the enamovirus grapevine enamovirus 1 (GEV-1, family *Luteoviridae*) [39,40]. QRV1 appears to have a monopartite (+)ssRNA genome similar to that of enamoviruses, but its sequence contig appeared to lack the 3′-terminal sequence region. During BLAST analyses, an additional short sequence contig (DN30870 _c0_g1_i1, 1118 nt), which is possibly the 3′-terminal region of the QRV1 genome that encodes a putative protein having a 53.4% identity to the coat protein (CP) readthrough domain (RTD) of an enamovirus (Accession No. YP_009249825.1) was also identified (Figure 3). Moreover, phylogenetic analysis based on RdRP (a putative P1-P2 fusion) sequence indicated close relatedness of QRV1 with enamoviruses (Figure 4); therefore, QRV1 is possibly a novel virus species in the genus *Enamovirus*. QRV2-encoded putative RdRP had a 48.3% identity with that of beihai narna-like virus 23, a monopartite narna-like virus [41]. The presence of a predicted single open reading frame (ORF) in its (+)ssRNA genome and phylogenetic relationship based on RdRP sequence (Figure 3 and Figure 4) further supports the view that QRV2 belongs to the narna-like virus group (a group of betanarnaviruses, sub-clade 1) [42]. QRV3 had a large ssRNA segment with negative-strand polarity and encoded a putative RdRP that had a 37.1% identity with that of apple rubbery wood virus 1 (ARWV-2), a rubodvirus with three (–)ssRNA segments belonging to the family *Phenuiviridae* [43,44]. A sequence contig, which is possibly a partial sequence of the additional segment of the QRV3 genome because it encoded a putative protein that had a 30.4% identity with a putative nucleocapsid protein of ARWV-2 (Accession No. QZW25191), was also identified (Figure 3). Phylogenetic analysis based on RdRP (L protein) sequence showed that QRV3 formed a clade with other rubodviruses (Figure 4), suggesting that QRV3 is a novel virus belonging to the genus *Rubodvirus*. QRV4 had a negative-strand genome (Figure 3), and its putative RdRP had a 22.2% identity with that of the yingvirus, Wuhan insect virus 15 (WhIV-15), a bipartite (–)ssRNA virus belonging to the family *Qinviridae*, which has divergent virus members that are usually identified in insects [41]. Moreover, identification of a sequence contig (DN12572_c0_g1_i1, 2628 nt), possibly a candidate for the second segment of QRV4, which encoded a putative protein having a 29.3% identity with a hypothetical protein of WhIV-15 (Accession No. YP_009342457) as well as its phylogenetic relatedness to members of *Qinviridae* based on RdRP sequence (Figure 3 and Figure 4) suggest that QRV4 is related to the yingvirus group.

Fifteen virus-related contigs representative of each viral genome or segment were selected for further analyses, considering their length (>2000 nt) and composing read number (>1000 reads) as well as their unrelatedness to bacterial viruses (Table 1 and Supplementary File S1). RT-PCR assays were then carried out for each total RNA sample using primers designed based on the sequences of the 15 virus-related sequences. All the tested viruses except for an umbravirus, IxYMV, were detected in the plant samples (Table 2). Notably, the majority of the plant samples contained multiple viruses (2–6 viruses); for example, the celery sample contained six viruses; the napa cabbage, bok choy, and watermelon samples each contained five viruses; and the radish-1 and radish-2 samples both contained four viruses (Table 2). Likewise, it was observed that many viruses were widely distributed among plant leaf samples. CMV had the highest prevalence, infecting the majority of plant samples (eight out of 11 samples). TuMV was detected in six samples, while BBWV-2 and ZYMV were detected in five samples. None of the samples, including those consisting of the same plant species, were found to have the same virus infection profile. 

### 3.3. Detection of Plant Viruses in the Fungal Strains

Representative leaf tissue of each sample was used as the source material for the isolation of fungal strains. A total of 241 fungal strains were isolated, with the number of fungal strains obtained largely differing among the leaf samples (Table 3). Unfortunately, fungus could not be isolated from the radish-1, radish-3, and watermelon samples due to bacterial contamination. After isolation, the fungal strains were maintained in the laboratory via periodic subcultures on PDA medium. To test for the presence of the abovementioned plant viruses in the fungal strains, total RNA was extracted from each fungal strain and subjected to RT-PCR assays and sequencing of the PCR products. Note that when the total RNA was extracted, the fungal strains had been subcultured at least six times. The selection of viruses tested for each fungal strain was based on the virus species that were observed to exist in the leaf sample from which the fungus was isolated. Out of 241 fungal strains, only 169 fungal strains were tested via RT-PCR; thus, a number of fungal strains were not tested, in particular, the fungi from the leaf sample batches from which a large number of fungal strains were obtained (see Table 3). 

**Figure 5:**
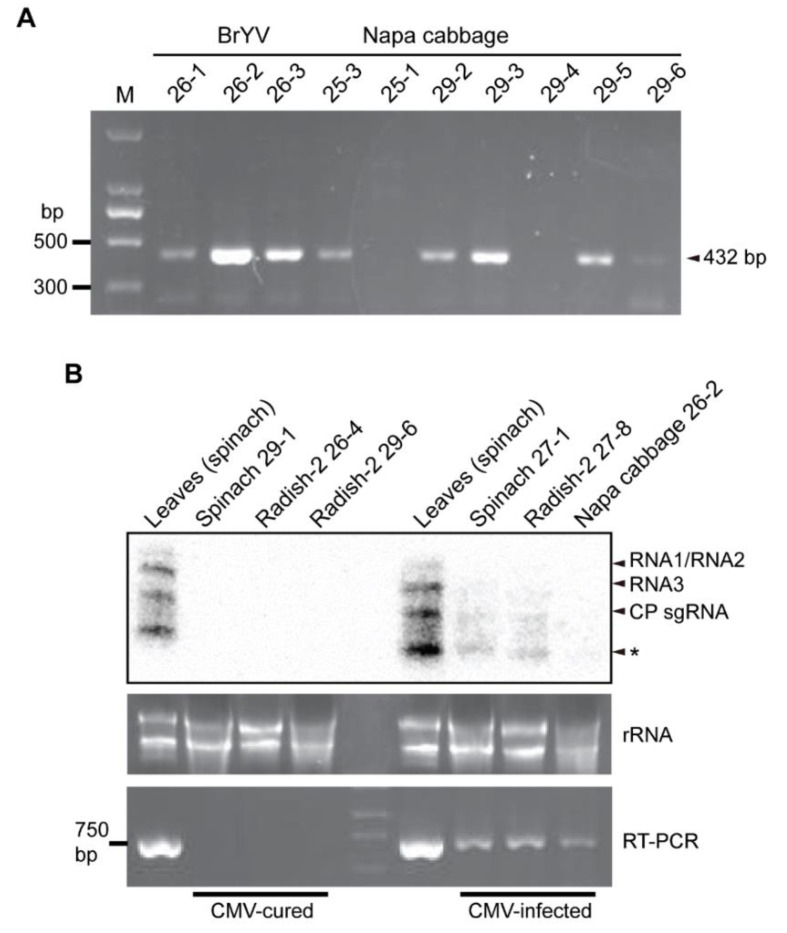
Detection of plant viruses in the fungal strains. (**A**) Nested RT-PCR detection of BrYV in the fungal strains isolated from the napa cabbage sample. (**B**) Detection of CMV in the fungal strains by RT-PCR and Northern blotting. * indicates undefined bands.

In the initial RT-PCR assay followed by sequencing of the PCR products, unspecific PCR bands were sometimes obtained; therefore, for each RT-PCR, if an expected size of PCR product was obtained, a second round of PCR was performed using nested primers. Nested RT-PCR and confirmation by sequencing detected the majority of plant virus and novel virus species in many of the fungal strains (Figure 5A and Supplementary Figure S2). The summary of the detection results showed that out of the 169 fungal strains tested, 89 strains (52.7%) representing fungi from all leaf sample batches were positive for the presence of viruses, with 25 fungal strains carrying multiple viruses (2–3 viruses; Table 3 and Supplementary File S2). Thus, around half of the fungal strains carried plant and novel viruses. Nine virus species, namely CMV, BBWV-2, ZYMV, TuMV, BrYV, QRV1, BnRV1, QRV3, QRV4, and one sub-viral RNA (TuYV-aRNA) belonging to eight virus genera (*Cucumovirus*, *Potyvirus*, *Polerovirus*, *Waikavirus*, *Rubodvirus*, *Qinvirus*, *Fabavirus*, and *Enamovirus*) were detected in the fungal strains, whereas KoMV, QRV2, and WMV were not detected in any of the fungal strains. CMV was most frequently detected in the fungal strains, with 38.4% prevalence (43 out of 112 strains tested), which is in accordance with the observation that CMV was present in the majority of the leaf samples (Table 2). This CMV strain had a 92% nucleotide and a 97% amino acid sequence identity with a CMV-Rs strain that was previously found in *R. solani* [22]. Moreover, analysis of the partial sequences of CMV RNA1 (1a coding region) and RNA3 (movement protein-coding region) showed nucleotide and amino acid sequence differences in RNA3 but not in RNA1 between the virus strains detected in the plant and fungal isolates (Supplementary Figure S3), suggesting the presence of natural virus variants or the occurrence of mutations in the fungal hosts. BBWV-2 was also detected in a relatively high number of fungi, but it had a lower prevalence (20.4%, 20 out of 98 strains tested). Among all viruses, BrYV had the highest prevalence (88.9%, 16 out of 18 strains tested). ZYMV, QRV3, QRV4, and TuYV-aRNA also had a relatively high prevalence (37.2, 50.0, 50.0, and 46.7%, respectively), while TuMV, QRV1, and BnRV1 had a low prevalence (5.9, 14.3, and 5.9%, respectively; Table 3). Interestingly, several fungal strains carried multiple viruses, given that most of the leaf samples contained multiple viruses. Nevertheless, fungi carrying only up to three viruses were found (Table 3 and Supplementary File S2), although many leaf samples contained 4–6 viruses (Table 2). In the majority of fungi isolated from the napa cabbage sample, multiple viruses were detected (16 out of 18 infected strains); all of them were from leaf samples that contained CMV and BrYV, with some fungal strains harbored three viruses with the addition of ZYMV or BBWV-2. A high proportion of fungal strains isolated from the celery sample, which contained ZYMV, QRV1, QRV3, and QRV4, also harbored multiple viruses (QRV3 and QRV4 or QRV1, QRV3, and ZYMV). In addition, ZYMV and CMV or BnRV1 were identified among fungal strains isolated from the bok choy sample.

Next, Northern blot analysis was carried out to examine plant virus accumulation levels in the fungal hosts. Northern blotting showed that the accumulation of CMV RNA was much lower relative to its accumulation in the plants (Figure 5B, CMV-infected). Moreover, the accumulation of other plant viruses in fungi could not be detected by Northern blotting, which is likely because virus accumulation was below the limit of detection level of Northern blotting. After continuous subculture of the fungal strains in the laboratory (more than six subcultures), many of the fungal strains were found to have lost the viruses (cured). For example, some fungal strains that were previously identified to carry CMV were free of CMV after prolonged maintenance of the fungal culture in the laboratory (Figure 5B, CMV-cured). This observation suggests that the presence of these viruses is not stable in fungal hosts that are artificially cultured under laboratory conditions.

### 3.4. Species Identification of the Fungal Strains Carrying Plant Viruses

**Figure 6:**
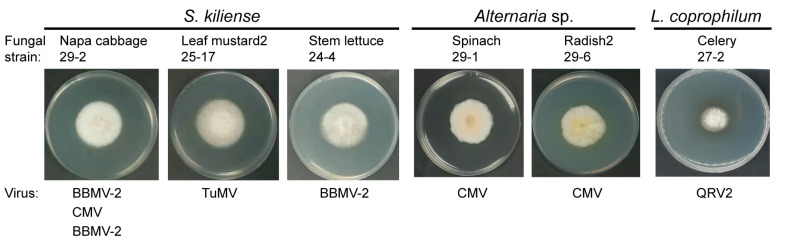
Phenotypic growth of representative fungal strains infected with plant viruses. The colonies were grown on PDA medium for 7 days and then photographed.

A total of 56 fungal strains carrying plant viruses were analyzed for species identification using DNA barcoding markers. Sequence analyses of ITS and/or TEF1α and BLAST searches against GenBank revealed that the fungal strains belong to three species groups (Supplementary File S3). According to ITS and TEF1α sequence identities by BLASTn search against ITS from fungi type and reference material (a RefSeq curated dataset) and nucleotide collection, respectively, the majority of the fungal strains (52 strains) were most likely to be *Sarocladium kiliense* (syn. *Acremonium kiliense*, order Hypocreales) (Supplementary File S4), while two strains were identified as *Lecanicillium coprophilum* (order Hypocreales; Supplementary File S5). Two other strains belong to the genus *Alternaria* (order Pleosporales, family Pleosporaceae), but their species classification was not clear (Supplementary File S6). *S. kiliense* was derived from all leaf samples and carried all 10 viruses detected. *L. coprophilum* was derived from the celery and radish-2 leaf samples and harbored QRV1 and BBWV-2, while *Alternaria* sp. was derived from the radish-2 and spinach leaf samples and harbored CMV (Supplementary File S2). Thus, it appears that *S. kiliense* is predominant among the fungal strains isolated, and there is no clear specificity between the plant viruses and their fungal hosts. Representative fungal colonies of *S. kiliense*, *L. coprophilum*, and *Alternaria* sp. cultured on PDA medium are presented in Figure 6. 

## 4. Discussion

Diverse viruses that infect fungi (fungal viruses or mycoviruses) have been identified in various groups of fungi [42,45,46]. An extracellular phase is generally absent in the life cycle of fungal viruses as many of them are usually transmitted vertically through sporulation and horizontally via hyphal fusion. Thus, unlike the majority of animal and plant viruses, many fungal viruses lack a capsid [45]. Nevertheless, many fungal viruses share genetic features with plant viruses, as indicated by their close taxonomic and phylogenetic relatedness, strikingly exemplified by alphapartitiviruses (segmented dsRNA viruses, family *Partitiviridae*), alphaflexiviruses (non-segmented (+)ssRNA viruses, family *Alpha-flexiviridae*), and endornaviruses (capsid-less (+)ssRNA viruses, family *Endornaviridae*) [11,47,48]. This suggests that horizontal transmission between plants and fungi plays an important role in the evolution of viruses. 

In this study, we identified ten genetically diverse viruses representing eight virus genera and six virus families carried by fungal strains isolated from the leaves of diseased plants. Notably, most of these viruses (potyviruses, luteoviruses, fabaviruses, and waikaviruses) are known to be vectored by insects, such as aphids and leafhoppers [9]. Thus, our results further revealed that many plant viruses could spread beyond their plant hosts and insect vectors. In our data, CMV, a cucumovirus (family *Bromoviridae*), was the most frequently detected in the fungal strains. This result seems plausible as the majority of the leaf samples were infected with CMV. CMV has the largest host range among plant viruses and is one of the most widespread plant viruses in agricultural fields [49]. As natural infection of CMV in *R. solani* was previously discovered [22], this study further revealed the wide spread of CMV to plant-associated fungi in the agroecosystem. In addition to CMV, TuMV, a potyvirus (family *Potyviridae*), and BBWV2, a fabavirus (family *Secoviridae*), were reported to be the predominant viruses infecting vegetable crops in China [50]. ZYMV, a potyvirus, is commonly detected in cucurbit plants in China and other countries [51,52,53,54], but in this study, we detected ZYMV in non-cucurbit plants. BrYV, a member of the tentative species of the genus *Polerovirus* (family *Solemoviridae*), has been reported to infect crucifer crops in China and Japan [55,56]. TuYV-aRNA has a high similarity to TuYV-aRNA previously found in weed (NCBI Accession No. QKG33160). Some subviral RNAs associated with poleroviruses, such as beet western yellows virus, are known to replicate independently but rely on poleroviruses as a helper for encapsidation and probably aphid transmission in the field [57,58]. Although TuYV-aRNA was found in the leaf mustard sample in this study, its potential helper virus, TuYV, was not found in the same sample. BnRV1, a waikavirus (family *Secoviridae*), was first identified in a transcriptome dataset of rapeseed [59]. QRV1 and QRV3 are most closely related to the viruses in the genera *Enamovirus* and *Rubodvirus*, respectively, virus genera that consist of plant-infecting members [60,61], suggesting that QRV1 and QRV3 are likely also plant-infecting viruses. QRV4 is most closely related to qinviruses, a divergent virus group that is usually found in insects [41,62,63]. Thus, it is not clear whether QRV4 is indeed a plant virus. However, an almost complete QRV4 genome sequence assembled from a relatively high number of reads was obtained from RNA-seq analysis of leaf samples, which may imply the multiplication of QRV4 in the plant host; however, it is also possible that QRV4 efficiently associated with leaf tissue, although it does not replicate in the plant cell. Taken together, except for the three novel viruses (QRV1, QRV3, and QRV4), these RNA viruses found in fungi were previously reported to widely infect and cause disease in varieties of crop plants; moreover, fungal viruses that are taxonomically related to these viruses have not so far been identified in fungi. Therefore, our findings represent naturally occurring acquisition of bona fide plant viruses in fungi during fungal colonialization of the plants. It is worth mentioning that a novel (−)ssRNA virus related to the multipartite phenui-like viruses (similar to QRV3) was identified in the shiitake mushroom (*Lentinula edodes*) [64], suggesting that the members of related virus group spread to both plant and fungal hosts.

The fungal strains in this study that were infected with plant viruses belonged to three fungal species, *S. kiliense*, *L. coprophilum,* and *Alternaria* sp., the majority being *S. kiliense*. Genus *Sarocladium* is comprised of a highly diverse group of fungi including plant pathogens, endophytes, saprobes, mycoparasites, and human opportunistic pathogens [65,66]. *S. kiliense* is mainly known as a human opportunistic pathogen [67,68,69,70], but it is also reported to be a plant pathogenic and endophytic fungus in some plant species [71,72,73,74,75]. A BLASTn search querying the ITS of *S. kiliense* strains isolated in this study against the nucleotide collection in GenBank yielded the majority of hits representing endophytes isolated from plant samples [76] (see Supplementary File S4). *L. coprophilum* was first identified from the fresh fecal matter of *Marmota monax* (groundhog) [77]. The species of *Lecanicillium* are known to be entomopathogens, mycoparasites, or endophytes [78,79]. *Alternaria* species are ubiquitous in the environment, with a number of species being major plant pathogens, saprophytes, endophytes, and opportunistic pathogens or allergens of humans [80,81,82]. As the leaf samples used in this study did not show any discernable symptoms of fungal disease, it is likely that the fungal strains isolated in this study are plant endophytic fungi. Unfortunately, due to the instability of the presence of viruses in the fungal strains (discussed below), the effect of viruses on the growth and morphology of the fungal host could not be examined in this study.

The finding of the common acquisition of plant viruses by plant-associated fungi highlights the possibility that fungi are involved in the epidemiology, emergence, and evolution of plant viruses. In an attempt to understand the epidemiology and emergence of plant viruses, intensive investigations have been conducted on the vector transmission and host range or reservoir of plant viruses in nature [7,83,84]; however, such investigations have not been extended to fungal species. Given the finding that plant viruses can multiply in fungi and be bidirectionally transmitted between fungi and plants, fungi could be considered potential biological vectors of plant viruses, a phenomenon parallel to plant virus transmission by insect vectors in a persistent-propagative manner [85]. In fact, a number of plant viruses belonging to the genera *Ophiovirus* (family *Ophioviridae*), *Varicosavirus* (family *Rhabdoviridae*), *Potexvirus* (family *Alphaflexiviridae*), and several genera in the family *Tombusviridae* are transmitted by the zoosporic soil-inhabiting fungi *Olpidium virulentus*, *O. brassicae*, and *O. brassicae* (family *Olpidiaceae*), although viruses do not multiply in these fungal vectors [8,86]. The biological vectors of many plant viruses are still unknown, and therefore, the involvement of various plant-associated fungi, particularly filamentous fungi, in plant virus transmission merits further investigation. Furthermore, our finding also suggests that aside from plants and arthropods, fungi could be the living sources or reservoirs of plant viruses in nature. In previous studies, we observed that in *R. solani*, CMV is horizontally transmitted by hyphal anastomosis but not vertically transmitted through basidiospores (sexual spores), while in *F. graminearum*, TMV is vertically transmitted through conidia (asexual spores) [19,22]. These observations suggest that for certain plant virus and fungal host combinations, plant viruses can be maintained in fungal populations in nature. In this study, we observed that after continuous subculture (more than six rounds of subculture) of the fungal strains in the laboratory, plant virus was not stable in the fungal host. It is possible that these plant viruses can not persistently exist in the fungal host. Another possibility is that the persistency of these plant viruses in these fungal strains requires specific conditions that are present during the natural fungal colonialization of the plant. Thus, fungal strains that are cultured in an artificial nutrient-rich medium under laboratory conditions may not support the existence of these plant viruses. We envisage that once the plant virus cross-infects a fungus, the virus may be transmitted among fungal strains and stably maintained in the fungal population. In this scenario, fungi serve as the living sources or reservoirs of plant viruses in nature. Meanwhile, fungi can also transmit plant viruses to the plant during the fungal colonialization of the plant. Moreover, plant viruses may also be indirectly transmitted from fungi to plants, for example, via insects, as many insects could consume both plants and fungi. Further investigation of cross-infection of plant viruses in fungi in agricultural and nonagricultural settings, including the examination of soil-borne fungi, would provide more insights into how fungi become integral components of plant virus ecology.

## Figures and Tables

**Table 1:** A list of plant viruses identified by RNA-seq.

Contig (DDBJ Accession No.)	Length (nt)	NGS Reads	BLASTn/BLASTx Results (nt/aa)					Genus (or Group) Tentative Virus Name
			Accession No.	E-Value	Identity ^a^	Segment/ Protein	Virus (Abbreviation)	
DN122318_c150_g1_i1 (LC726798)	3363	5,854,518	AB179764.1/ QFZ79258.1	0/ 0	97.0/ 99.5	RNA1/ protein 1a	Cucumber mosaic virus (CMV) ^b^	*Cucumovirus*
DN111361_c93_g1_i1 (LC726799)	2866	1,863,038	AF314188.1/ Q86783.1	0/ 0	98.6/ 99.4	RNA2/ RdRP		
DN124624_c138_g1_i1 (LC726800)	1367	741,776	KP710853.1/ ACB56605.1	0/ 0	98.5/ 99.6	RNA3/ MP		
DN133764_c33_g1_i1 (LC726792)	5901	313,982	KC790225.1/ AGO58930.1	0/ 0	97.8/ 99.5	RNA1/ polyprotein	Broad bean wilt virus 2 (BBWV-2)	*Fabavirus*
DN132884_c28_g1_i1 (LC726793)	3653	253,794	AB746939.1/ AZF99051.1	0/ 0	92.1/ 97.3	RNA2/ polyprotein		
DN132743_c15_g1_i1 (LC726785)	9598	149,164	KX421104.1/ ARN61640.1	0/ 0	98.0/ 99.0	– ^e^/ polyprotein	Zucchini yellow mosaic virus (ZYMV)	*Potyvirus*
DN97429_c17_g1_i1 (LC726783)	10,344	133,131	MF085000/ AWB03290.1	0/ 0	98.3/ 99.2	–/ polyprotein	Papaya ringspot virus (PRSV)	
DN133242_c23_g1_i2 ^c^ (LC726791)	6247	126,843	KC119188.1/ AGD80385.1	0/ 0	97.2/ 99.0	–/ polyprotein	Turnip mosaic virus (TuMV)/	
DN105402_c0_g2_i1 (LC726786)	9451	43,227	MW961163.1/ QJW82783.1	0/ 0	78.4/ 88.1	–/ polyprotein	Konjac mosaic virus (KoMV)	
DN134304_c2_g2_i7 (LC726784)	9894	1267	MN914159.1/ QIQ08170.1	0/ 0	94.2/ 97.6	–/ polyprotein	Watermelon mosaic virus (WMV)	
DN71321_c0_g2_i1 (LC726782)	12,267	3976	NC_040586.1/ YP_009552078.1	0/ 0	98.9/ 99.5	–/ replicasepolyprotein	Brassica napus RNA virus 1 (BnRV1)	*Waikavirus*
DN128817_c10_g1_i1 ^c^ (LC726796)	4345	62,121	KT946712.1/ AMR60139.1	0/ 0	82.5/ 91.5	–/ RdRP	Ixeridium yellow mottle virus 2 (IxYMV-2)	*Umbravirus*
DN134266_c8_g3_i2 ^d^ (LC726802)	2740	15,684	LC428358.1/ BBG75734.1	0/ 0	96.9/ 97.3	–/ P1	Brassica yellows virus (BrYV)/	*Polerovirus*
DN134266_c8_g2_i1 ^d^ (LC726803)	1175	10,234	LC428358.1/ BBG75771.1	0/ 0	98.4/ 98.7	–/ CP		
DN130249_c0_g1_i1 (LC726801)	2816	1920	MN497834.1/ QKG33160.1	0/ 0	95.1/ 97.4	–/ RdRP	Turnip yellows virus-associated RNA (TuYV-aRNA)	Unassigned polerovirus associated RNA
DN120852_c1_g1_i1 ^d^ (LC726794)	4806	12,352	– ^e^/ QTF33728.1	–/ 0	–/ 64.1	–/ RdRP	Grapevine enamovirus 1	*Enamovirus* Qingdao RNA virus 1 (QRV1) ^f^
DN30870_c0_g1_i1 ^d^ (LC726795)	1118	2961	–/ YP_009249825.1	–/ 7 × 10^84^	–/ 53.4	–/ CP read-through domain	Alfalfa enamovirus 1	
DN131759_c2_g1_i1 (LC726797)	3462	9913	–/ APG77086.1	–/ 0	–/ 48.3	–/ RdRP	Beihai narna-like virus 23	narna-like virus Qingdao RNA virus 2 (QRV2) ^f^
DN118628_c0_g1_i1 (LC726787)	7275	3409	–/ QUV77595.1	–/ 0	–/ 37.1	RNA1/ RdRP	Apple rubbery wood virus 1	*Rubodvirus* Qingdao RNA virus 3 (QRV3) ^f^
DN109466_c0_g1_i1 (LC726788)	1433	235	–/ AYS94195.1	–/ 8 ×^−^10^21^	–/ 33.5	RNA2/ putative CP		
DN109396_c0_g1_i1 (LC726789)	6279	2527	–/ NC_033490.1	–/ 9 ×^−^10^59^	–/ 22.2	RNA1/ RdRP	Wuhan insect virus 15	*Qinvirus* Qingdao RNA virus 4 (QRV4) ^f^
DN12572_c0_g1_i1 (LC726790)	2628	1611	–/ YP_009342457.1	–/ 3 × 10^07^	–/ 29.3	RNA2/ hypothetical protein		

**^a^** Nucleotide/amino acid sequence identity (%). **^b^** The predominant sequence contigs derived from CMV subgroup I were selected for subsequent analyses, whereas the virus of another CMV subgroup (II) was also detected in the RNA pool (see Supplementary File S1). **^c^** Lacking the sequences regions of the 3′ terminal genomes. **^d^** These sequence contigs were likely derived from a polerovirus and a noble enamovirus, respectively. **^e^** No or very week hits/not applicable. **^f^** A member of the putative novel virus species.

**Table 2:** RT-PCR detection of viruses in leaf samples.

Leaf Samples	Viruses													
	CMV	BBWV-2	ZYMV	PSRV	TuMV	KoMV	WMV	BnRV1	BrYV	TuYV-aRNA	QRV1	QRV2	QRV3	QRV4
Spinach	**+**													
Leaf mustard-1					**+**					**+**				
Leaf mustard-2	**+**				**+**			**+**						
Radish-1	**+**	**+**	**+**		**+**									
Radish-2	**+**	**+**						**+**				**+**		
Radish-3	**+**				**+**		**+**							
Napa cabbage	**+**	**+**	**+**		**+**				**+**					
Bok choy	**+**		**+**		**+**		**+**	**+**						
Celery		**+**	**+**			**+**					**+**		**+**	**+**
Stem lettuce		**+**									**+**			
Watermelon	**+**		**+**	**+**			**+**	**+**						

+: positive detection by RT-PCR.

**Table 3:** RT-PCR detection of plant viruses in the fungal strains.

Leaf Samples	No. of Fungi		No. of Fungal Strains with Virus Infection															
	Isol.	Tested	CMV	BBWV-2	ZYMV	PSRV	TuMV	KoMV	WMV	BnRV1	BrYV	TuYV-aRNA	QRV1	QRV2	QRV3	QRV4	Total	Mixed Infection
Spinach	21	21	17														17	
Leaf mustard-1	21	15					0					7					7	
Leaf mustard-2	39	18	0				4			0							4	
Radish-1	0	0																
Radish-2	55	38	4	11						0				0			14	1
Radish-3	0	0																
Napa cabbage	18	18	16	2	4		0				16						18	16
Bok choy	17	17	6		8		0		0	1							13	2
Celery	8	8		0	4			0					4		4	4	8	5
Stem lettuce	62	34		7									2				8	1
Watermelon	0	0																
Total	241	169	43	20	16		4			1	16	7	6		4	4	89	25
Prevalence (%)			38.4	20.4	37.2		5.9			5.9	88.9	46.7	14.3		50	50		

## Data Availability

The raw data supporting the conclusions of this article will be made available by the authors without undue reservation.

## Supplementary Materials

The following are available online at https://www.mdpi.com/article/10.3390/v14102279/s1, Figure S1. Proportion of the reads that were assembled as the virus-related sequence contigs. Figure S2. Nested RT-PCR detection of plant viruses in the fungal strains isolated from the leaf samples. Figure S3. Alignment of partial nucleotide and amino acid sequences of CMV RNA1 (1a coding region) and CMV RNA3 (movement protein-coding region) derived from virus strains detected in plants and fungi. Table S1. A list of primers used in this study. Supplementary File S1. BLAST search results of the assembled sequence contigs as queries against virus databases. Supplementary File S2. A list of fungal strains carrying plant viruses. Supplementary File S3. ITS and TEF1a sequences of fungal strains carrying plant viruses. Supplementary File S4. BLASTn search results querying the ITS and TEF1a sequences of *S. kiliense* strain against fungi type and reference material (a RefSeq curated dataset) and nucleotide collection in GenBank. Supplementary File S5. BLASTn search results querying the ITS and TEF1a sequences of *Lecanicillium coprophilum* strain against fungi type and reference material (a RefSeq curated dataset) and nucleotide collection in GenBank. Supplementary File S6. BLASTn search results querying the ITS and TEF1a sequences of *Alternaria* strain against fungi type and reference material (a RefSeq curated dataset) and nucleotide collection in GenBank.
