# Supplementary material for: Common but Nonpersistent Acquisitions of Plant Viruses by Plant-Associated Fungi
Source: Viruses. 2022 Oct 17;14(10):2279. doi: 10.3390/v14102279 (PMC9611831; doi:10.3390/v14102279)
Supplement: Supplementary file 1 [file viruses-14-02279-s001.zip › viruses-1937532-supplementary/Supplementary Figure S2.pdf]

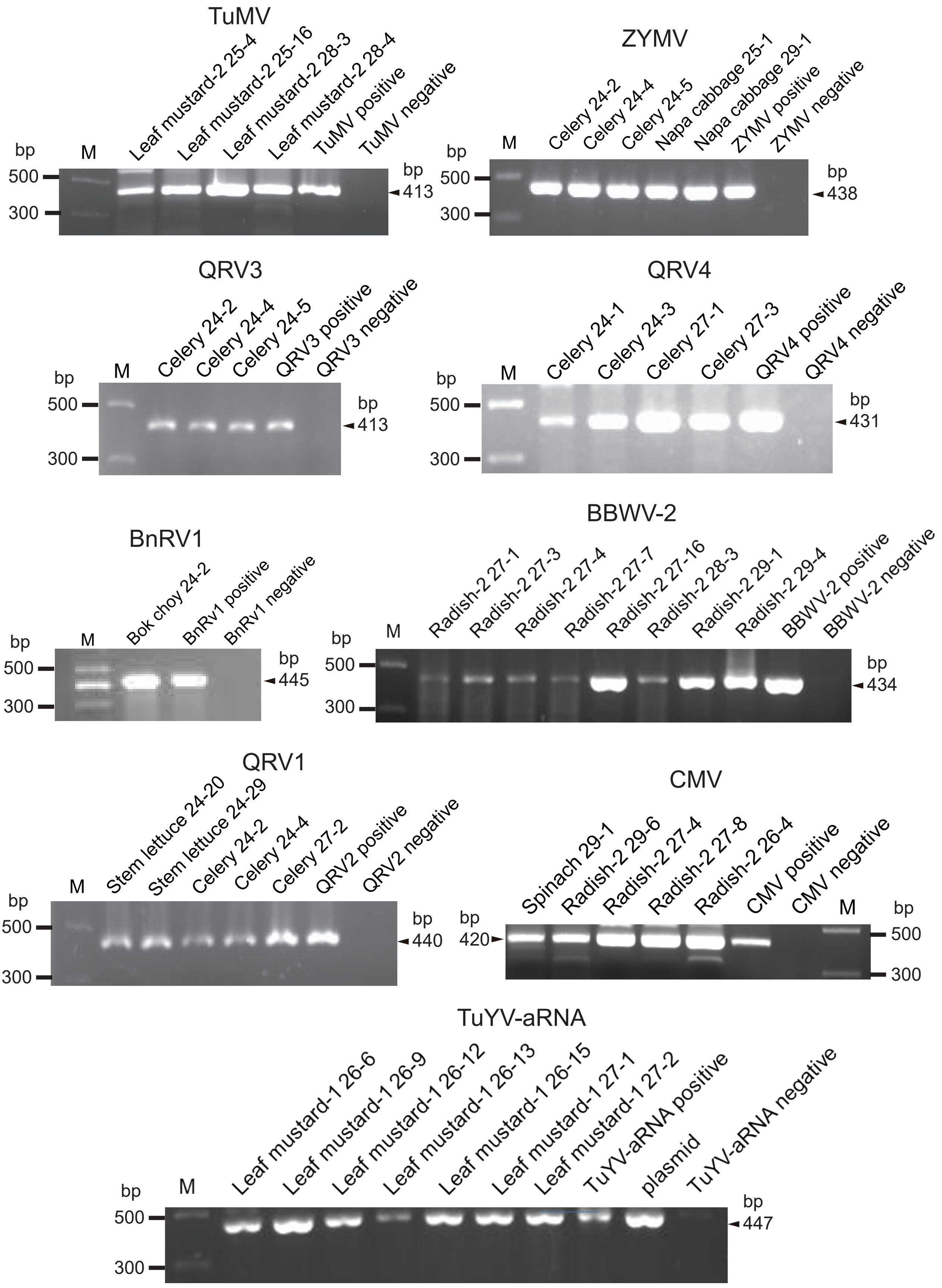

Figure S2. Nested RT-PCR detection of plant viruses in the fungal strains isolated from the leaf samples.
