# Supplementary material for: Common but Nonpersistent Acquisitions of Plant Viruses by Plant-Associated Fungi
Source: Viruses. 2022 Oct 17;14(10):2279. doi: 10.3390/v14102279 (PMC9611831; doi:10.3390/v14102279)
Supplement: Supplementary file 1 [file viruses-14-02279-s001.zip › viruses-1937532-supplementary/Supplementary Figure S3.pdf]

CMV RNA1 (1a)

```
CLUSTAL format alignment by MAFFT (v7.505) samples, plant:sPI-13;
                                                 fungus:Radish-2 27-4

                GGCTTTAGAGGATTATGTG (CMV-CF:5'primer)
plant           ggctttagaggactgtgtgaagcgatgaacgctcacggaaccactattttgaagggaacg
fungus          ----------------------------------------------------agggaacg
                                                                    ********

plant           atgatgttcgatggtgctatgatgtttgacgaccaaggtgtaatacccgaacttaactgt
fungus          atgatgttcgatggtgctatgatgtttgacgaccaaggtgtaatacccgaacttaactgt
                ************************************************************

plant           cagtggagaaagatcaggagtgctttctctgaaactgaagacgtcacaccgctgactgag
fungus          cagtggagaaagatcaggagtgctttctctgaaactgaagacgtcacaccgctgactgag
                ************************************************************

plant           aaactcaattccacaatattctcccgcgtgcgtaaattcaagactatggtggctttcgat
fungus          aaactcaattccacaatattctcccgcgtgcgtaaattcaagactatggtggctttcgat
                ************************************************************

plant           tttatcaacgagtctactatgtcttatgtacatgattgggagaatataaaatcttttctc
fungus          tttatcaacgagtctactatgtcttatgtacatgattgggagaatataaaatcttttctc
                ************************************************************

plant           acggatcagacttattcataccgagggatgacctatggtattgagcggtgcgttattcac
fungus          acggatcagacttattcataccgagggatgacctatggtattgagcggtgcgttattcac
                ************************************************************

plant           gctggtattatgacgtataagatcattggtgtacctgggatgtgcccacccgaactca
fungus          gctggtattatgacgtataagatcattggtgtacctgggatatgcccacccgaactca
                *****************************************.****************
                                                        TATGCCCACCCGAACTCA
                                                         (CMV-CR:3'primer)
```

CMV RNA3 (MP)

```
CLUSTAL format alignment by MAFFT (v7.505) Samples, plant:Radish-2;
                                                 fungus:Radish-2 27-4


                             AGGCATGGCTTTCCAAGGTAC (CM-R3-119-F:5'primer)
Plants                                 ttccaaggtaccagcaggactttaacccaacagtcct
fungus                                 ttccaaggtaccagcaggactttaacccaacagtcct
                                       *************************************

Plants          cagcggctacgtctgacgaacttcaaaagatattatttagccctgatgccatcaagaaaa
fungus          cagcggctacgtctgacgatcttcaaaagatattatttagccctgaagccattaagaaaa
                ******************* ************************** *****.*******

Plants          tggctgctgagtgtgacctaggccggcatcattggatgcgtgctgataatgcgatttcag
fungus          tggctactgagtgtgacctaggccggcatcattggatgcgcgctgataatgctatttcag
                *****.**********************************.*********** *******

Plants          tccggcccctcgttcccgaagtaacccacggtcgtattgcttccttctttaagtctggat
fungus          tccggcccctcgttcccgaagtaacccacggtcgtattgcttccttctttaagtctggat
                ************************************************************

Plants          atgatgctggtgaattgtgctctaaaggatacatgagcgtccctcaagtgttgtgtgctg
fungus          atgatgttggtgaattatgctcaaaaggatacatgagtgtccctcaagtgttatgtgctg
                ******.*********.***** **************.**************.*******

Plants          ttactcgaacagtctccactgatgctgaagggtctctgagaatttacctagctgatttag
fungus          ttactcgaacagtttccactgatgctgaagggtctttgagaatttacttagctgatctgg
                *************.*********************.***********.********.*.*

Plants          gcgataaggagctatctcccatagataagcagtgcgtcacattacataaccatgatcttc
fungus          gcgacaaggagttatctcccatagatgggcaatgcgtttcgttacataaccatgatcttc
                ****.******.**************..***.*****. *.*******************

Plants          ccgctttagtgtctttccaaccgacgtatgattgtcccatggagacagttgggaatcgga
fungus          ccgctttggtgtctttccaaccgacgtatgattgtcctatggaaacagttgggaatcgta
                *******.*****************************.*****.************** *

Plants          agcggtgttttgcagttgtcatcgaaagacatggttatattgggtataccggcaccacag
fungus          agcggtgttttgctgtcgttatcgaaagacatggttacattgggtataccggtaccacag
                ************* **.**.*****************.**************.*******

Plants          ctagcgtgtgtagtaattggcaagcacggttttcttctaagaataacaactacactcata
fungus          ctagcgtgtgtagtaattggcaagcaaggttttcatccaagaataacaactacactcata
                ************************** ******* **.**********************


Plants          tcgcagctgggaagactctagtactgccattcaacagattagctgagcaaacgaaaaccc
fungus          tcgcagctgggaagactctagtactgcc--------------------------------
                ****************************
                                                         GCTGAGCAAACGAAACCGTCAG
                                                         (CM-R3-769-R :3'primer)

CLUSTAL format alignment by MAFFT (v7.505)

Plants       ...SSAATSDELQKILFSPDAIKKMAAECDLGRHHWMRADNAISVRPLVPEVTHGRIASFFKS
fungus       ...SSAATSDDLQKILFSPEAIKKMATECDLGRHHWMRADNAISVRPLVPEVTHGRIASFFKS
                *******:********:******:************************************

Plants          GYDAGELCSKGYMSVPQVLCAVTRTVSTDAEGSLRIYLADLGDKELSPIDKQCVTLHNHD
fungus          GYDVGELCSKGYMSVPQVLCAVTRTVSTDAEGSLRIYLADLGDKELSPIDGQCVSLHNHD
                ***.********************************************** ***:*****

Plants          LPALVSFQPTYDCPMETVGNRKRCFAVVIERHGYIGYTGTTASVCSNWQARFSSKNNNYTHIAAGKTLV...
fungus          LPALVSFQPTYDCPMETVGNRKRCFAVVIERHGYIGYTGTTASVCSNWQARFSSKNNNYTHIAAGKTLV...
                *********************************************************************
```

Figure S3. Alignment of partial nucleotide and amino acid sequences of CMV RNA1 (1a coding region) and CMV RNA3 (movement protein-coding region) derived from virus strains detected in plants and fungi.
