# Supplementary material for: Common but Nonpersistent Acquisitions of Plant Viruses by Plant-Associated Fungi
Source: Viruses. 2022 Oct 17;14(10):2279. doi: 10.3390/v14102279 (PMC9611831; doi:10.3390/v14102279)
Supplement: Supplementary file 1 [file viruses-14-02279-s001.zip › viruses-1937532-supplementary/Supplementary File S3.pdf]

Sarocladium kiliense

ITS sequences
>Bok choy 24-10
TTGTGAACATACCTATCGTTCCCTCGGCGGGCTCAGCGCGCGGTGCCTCCGGGCTCCGGGCGTCCGCCGGGGA
CAACCAAACTCTGATTTTATTGTGAATCTCTGAGGGGCGAAAGCCCGACAACAAAATGAATCAAAACTTTCAA
CAACGGATCTCTTGGCTCTGGCATCGATGAAGAACGCAGCGAAATGCGATAAGTAATGTGAATTGCAGAATTC
AGTGAATCATCGAATCTTTGAACGCACATTGCGCCCGCCGGCACTCCGGCGGGCATGCCTGTCCGAGCGTCAT
TTCAACCCTCAGGACCCCCTTTCGGGGGGGACCTGGTGCTGGGGATCAGCGGCCTCCGGGCCCCTGTCCCCCA
AATTGAGTGGCGGTCGCGCCGCAGCCTCCCCTGCGTAGTAGCACACCTCGCACCGGAGAGCGGCTCGGCCACG
CCGTGAAACCCCCAATTTTTTAAGGTTGACCTCGGATCAGGTAGGAATACCCGCTGAACTTAAGCATAT
>Bok choy 24-11
TTGTGAACATACCTATCGTTCCCTCGGCGGGCTCAGCGCGCGGTGCCTCCGGGCTCCGGGCGTCCGCCGGGGA
CAACCAAACTCTGATTTTATTGTGAATCTCTGAGGGGCGAAAGCCCGACAACAAAATGAATCAAAACTTTCAA
CAACGGATCTCTTGGCTCTGGCATCGATGAAGAACGCAGCGAAATGCGATAAGTAATGTGAATTGCAGAATTC
AGTGAATCATCGAATCTTTGAACGCACATTGCGCCCGCCGGCACTCCGGCGGGCATGCCTGTCCGAGCGTCAT
TTCAACCCTCAGGACCCCCTTTCGGGGGGGACCTGGTGCTGGGGATCAGCGGCCTCCGGGCCCCTGTCCCCCA
AATTGAGTGGCGGTCGCGCCGCAGCCTCCCCTGCGTAGTAGCACACCTCGCACCGGAGAGCGGCTCGGCCACG
CCGTGAAACCCCCAATTTTTTAAGGTTGACCTCGGATCAGGTAGGAATACCCGCTGAACTTAAGCATAT
>Bok choy 24-12
TTGTGAACATACCTATCGTTCCCTCGGCGGGCTCAGCGCGCGGTGCCTCCGGGCTCCGGGCGTCCGCCGGGGA
CAACCAAACTCTGATTTTATTGTGAATCTCTGAGGGGCGAAAGCCCGACAACAAAATGAATCAAAACTTTCAA
CAACGGATCTCTTGGCTCTGGCATCGATGAAGAACGCAGCGAAATGCGATAAGTAATGTGAATTGCAGAATTC
AGTGAATCATCGAATCTTTGAACGCACATTGCGCCCGCCGGCACTCCGGCGGGCATGCCTGTCCGAGCGTCAT
TTCAACCCTCAGGACCCCCTTTCGGGGGGGACCTGGTGCTGGGGATCAGCGGCCTCCGGGCCCCTGTCCCCCA
AATTGAGTGGCGGTCGCGCCGCAGCCTCCCCTGCGTAGTAGCACACCTCGCACCGGAGAGCGGCTCGGCCACG
CCGTGAAACCCCCAATTTTTTAAGGTTGACCTCGGATCAGGTAGGAATACCCGCTGAACTTAAGCATAT
>Bok choy 24-13
TTGTGAACATACCTATCGTTCCCTCGGCGGGCTCAGCGCGCGGTGCCTCCGGGCTCCGGGCGTCCGCCGGGGA
CAACCAAACTCTGATTTTATTGTGAATCTCTGAGGGGCGAAAGCCCGACAACAAAATGAATCAAAACTTTCAA
CAACGGATCTCTTGGCTCTGGCATCGATGAAGAACGCAGCGAAATGCGATAAGTAATGTGAATTGCAGAATTC
AGTGAATCATCGAATCTTTGAACGCACATTGCGCCCGCCGGCACTCCGGCGGGCATGCCTGTCCGAGCGTCAT
TTCAACCCTCAGGACCCCCTTTCGGGGGGGACCTGGTGCTGGGGATCAGCGGCCTCCGGGCCCCTGTCCCCCA
AATTGAGTGGCGGTCGCGCCGCAGCCTCCCCTGCGTAGTAGCACACCTCGCACCGGAGAGCGGCTCGGCCACG
CCGTGAAACCCCCAATTTTTTAAGGTTGACCTCGGATCAGGTAGGAATACCCGCTGAACTTAAGCATAT
>Celery 24-1
TTGTGAACATACCTATCGTTCCCTCGGCGGGCTCAGCGCGCGGTGCCTCCGGGCTCCGGGCGTCCGCCGGGGA
CAACCAAACTCTGATTTTATTGTGAATCTCTGAGGGGCGAAAGCCCGACAACAAAATGAATCAAAACTTTCAA
CAACGGATCTCTTGGCTCTGGCATCGATGAAGAACGCAGCGAAATGCGATAAGTAATGTGAATTGCAGAATTC
AGTGAATCATCGAATCTTTGAACGCACATTGCGCCCGCCGGCACTCCGGCGGGCATGCCTGTCCGAGCGTCAT
TTCAACCCTCAGGACCCCCTTTCGGGGGGGACCTGGTGCTGGGGATCAGCGGCCTCCGGGCCCCTGTCCCCCA
AATTGAGTGGCGGTCGCGCCGCAGCCTCCCCTGCGTAGTAGCACACCTCGCACCGGAGAGCGGCTCGGCCACG
CCGTGAAACCCCCAATTTTTTAAGGTTGACCTCGGATCAGGTAGGAATACCCGCTGAACC
>Celery 24-2
TTGTGAACATACCTATCGTTCCCTCGGCGGGCTCAGCGCGCGGTGCCTCCGGGCTCCGGGCGTCCGCCGGGGA
CAACCAAACTCTGATTTTATTGTGAATCTCTGAGGGGCGAAAGCCCGACAACAAAATGAATCAAAACTTTCAA
CAACGGATCTCTTGGCTCTGGCATCGATGAAGAACGCAGCGAAATGCGATAAGTAATGTGAATTGCAGAATTC
AGTGAATCATCGAATCTTTGAACGCACATTGCGCCCGCCGGCACTCCGGCGGGCATGCCTGTCCGAGCGTCAT
TTCAACCCTCAGGACCCCCTTTCGGGGGGGACCTGGTGCTGGGGATCAGCGGCCTCCGGGCCCCTGTCCCCCA
AATTGAGTGGCGGTCGCGCCGCAGCCTCCCCTGCGTAGTAGCACACCTCGCACCGGAGAGCGGCTCGGCCACG
CCGTGAAACCCCCAATTTTTTAAGGTTGACCTCGGATCAGGTAGGAATACCCGCTGAACTTAAG
>Celery 24-4
TTGTGACATACCTATCGTTCCCTCGGCGGGCTCAGCGCGCGGTGCCTCCGGGCTCCGGGCGTCCGCCGGGGAC
AACCAAACTCTGATTTTATTGTGAATCTCTGAGGGGCGAAAGCCCGACAACAAAATGAATCAAAACTTTCAAC
AACGGATCTCTTGGCTCTGGCATCGATGAAGAACGCAGCGAAATGCGATAAGTAATGTGAATTGCAGAATTCA
GTGAATCATCGAATCTTTGAACGCACATTGCGCCCGCCGGCACTCCGGCGGGCATGCCTGTCCGAGCGTCATT
TCAACCCTCAGGACCCCCTTTCGGGGGGGACCTGGTGCTGGGGATCAGCGGCCTCCGGGCCCCTGTCCCCCAA

ATTGAGTGGCGGTCGCGCCGCAGCCTCCCCTGCGTAGTAGCACACCTCGCACCGGAGAGCGGCTCGGCCACGC
CGTGAAACCCCCAATTTTTTAAGGTTGACCTCGGATCAGGTAGGAATACCCGCTGAACTTAAGCA
>Celery 24-5
TTGTGAACATACCTATCGTTCCCTCGGCGGGCTCAGCGCGCGGTGCCTCCGGGCTCCGGGCGTCCGCCGGGGA
CAACCAAACTCTGATTTTATTGTGAATCTCTGAGGGGCGAAAGCCCGACAACAAAATGAATCAAAACTTTCAA
CAACGGATCTCTTGGCTCTGGCATCGATGAAGAACGCAGCGAAATGCGATAAGTAATGTGAATTGCAGAATTC
AGTGAATCATCGAATCTTTGAACGCACATTGCGCCCGCCGGCACTCCGGCGGGCATGCCTGTCCGAGCGTCAT
TTCAACCCTCAGGACCCCCTTTCGGGGGGGACCTGGTGCTGGGGATCAGCGGCCTCCGGGCCCCTGTCCCCCA
AATTGAGTGGCGGTCGCGCCGCAGCCTCCCCTGCGTAGTAGCACACCTCGCACCGGAGAGCGGCTCGGCCACG
CCGTGAAACCCCCAATTTTTTAAGGTTGACCTCGGATCAGGTAGGAATACCCGCTGAACTTAAGCA
>Celery 27-3
TTGTGAACATACCTATCGTTCCCTCGGCGGGCTCAGCGCGCGGTGCCTCCGGGCTCCGGGCGTCCGCCGGGGA
CAACCAAACTCTGATTTTATTGTGAATCTCTGAGGGGCGAAAGCCCGACAACAAAATGAATCAAAACTTTCAA
CAACGGATCTCTTGGCTCTGGCATCGATGAAGAACGCAGCGAAATGCGATAAGTAATGTGAATTGCAGAATTC
AGTGAATCATCGAATCTTTGAACGCACATTGCGCCCGCCGGCACTCCGGCGGGCATGCCTGTCCGAGCGTCAT
TTCAACCCTCAGGACCCCCTTTCGGGGGGGACCTGGTGCTGGGGATCAGCGGCCTCCGGGCCCCTGTCCCCCA
AATTGAGTGGCGGTCGCGCCGCAGCCTCCCCTGCGTAGTAGCACACCTCGCACCGGAGAGCGGCTCGGCCACG
CCGTGAAACCCCCAATTTTTTAAGGTTGACCTCGGATCAGGTAGGAATACCCGCTGAACTTAAGCATAT
>Leaf mustard-1 26-6
TACCTATCGTTCCCTCGGCGGGCTCAGCGCGCGGTGCCTCCGGGCTCCGGGCGTCCGCCGGGGACAACCAAAC
TCTGATTTTATTGTGAATCTCTGAGGGGCGAAAGCCCGACAACAAAATGAATCAAAACTTTCAACAACGGATC
TCTTGGCTCTGGCATCGATGAAGAACGCAGCGAAATGCGATAAGTAATGTGAATTGCAGAATTCAGTGAATCA
TCGAATCTTTGAACGCACATTGCGCCCGCCGGCACTCCGGCGGGCATGCCTGTCCGAGCGTCATTTCAACCCT
CAGGACCCCCTTTCGGGGGGGACCTGGTGCTGGGGATCAGCGGCCTCCGGGCCCCTGTCCCCCAAATTGAGTG
GCGGTCGCGCCGCAGCCTCCCCTGCGTAGTAGCACACCTCGCACCGGAGAGCGGCTCGGCCACGCCGTGAAAC
CCCCAATTTTTTAAGGTTGACCTCGGATCAGGTAGGAATACCCGCTGAACTTAAGCATAT
>Leaf mustard-1 26-12
TTGTGAACATACCTATCGTTCCCTCGGCGGGCTCAGCGCGCGGTGCCTCCGGGCTCCGGGCGTCCGCCGGGGA
CAACCAAACTCTGATTTTATTGTGAATCTCTGAGGGGCGAAAGCCCGACAACAAAATGAATCAAAACTTTCAA
CAACGGATCTCTTGGCTCTGGCATCGATGAAGAACGCAGCGAAATGCGATAAGTAATGTGAATTGCAGAATTC
AGTGAATCATCGAATCTTTGAACGCACATTGCGCCCGCCGGCACTCCGGCGGGCATGCCTGTCCGAGCGTCAT
TTCAACCCTCAGGACCCCCTTTCGGGGGGGACCTGGTGCTGGGGATCAGCGGCCTCCGGGCCCCTGTCCCCCA
AATTGAGTGGCGGTCGCGCCGCAGCCTCCCCTGCGTAGTAGCACACCTCGCACCGGAGAGCGGCTCGGCCACG
CCGTGAAACCCCCAATTTTTTAAGGTTGACCTCGGATCAGGTAGGAATACCCGCTGAACTTAAGCATAT
>Leaf mustard-1 26-13
TTGTGAACATACCTATCGTTCCCTCGGCGGGCTCAGCGCGCGGTGCCTCCGGGCTCCGGGCGTCCGCCGGGGA
CAACCAAACTCTGATTTTATTGTGAATCTCTGAGGGGCGAAAGCCCGACAACAAAATGAATCAAAACTTTCAA
CAACGGATCTCTTGGCTCTGGCATCGATGAAGAACGCAGCGAAATGCGATAAGTAATGTGAATTGCAGAATTC
AGTGAATCATCGAATCTTTGAACGCACATTGCGCCCGCCGGCACTCCGGCGGGCATGCCTGTCCGAGCGTCAT
TTCAACCCTCAGGACCCCCTTTCGGGGGGGACCTGGTGCTGGGGATCAGCGGCCTCCGGGCCCCTGTCCCCCA
AATTGAGTGGCGGTCGCGCCGCAGCCTCCCCTGCGTAGTAGCACACCTCGCACCGGAGAGCGGCTCGGCCACG
CCGTGAAACCCCCAATTTTTTAAGGTTGACCTCGGATCAGGTAGGAATACCCGCTGAAC
>Leaf mustard-1 26-15
TTGTGAACATACCTATCGTTCCCTCGGCGGGCTCAGCGCGCGGTGCCTCCGGGCTCCGGGCGTCCGCCGGGGA
CAACCAAACTCTGATTTTATTGTGAATCTCTGAGGGGCGAAAGCCCGACAACAAAATGAATCAAAACTTTCAA
CAACGGATCTCTTGGCTCTGGCATCGATGAAGAACGCAGCGAAATGCGATAAGTAATGTGAATTGCAGAATTC
AGTGAATCATCGAATCTTTGAACGCACATTGCGCCCGCCGGCACTCCGGCGGGCATGCCTGTCCGAGCGTCAT
TTCAACCCTCAGGACCCCCTTTCGGGGGGGACCTGGTGCTGGGGATCAGCGGCCTCCGGGCCCCTGTCCCCCA
AATTGAGTGGCGGTCGCGCCGCAGCCTCCCCTGCGTAGTAGCACACCTCGCACCGGAGAGCGGCTCGGCCACG
CCGTGAAACCCCCAATTTTTTAAGGTTGACCTCGGATCAGGTAGGAATACCCGCTGAACTTAAG
>Leaf mustard-2 25-4
TACCTATCGTTCCCTCGGCGGGCTCAGCGCGCGGTGCCTCCGGGCTCCGGGCGTCCGCCGGGGACAACCAAAC
TCTGATTTTATTGTGAATCTCTGAGGGGCGAAAGCCCGACAACAAAATGAATCAAAACTTTCAACAACGGATC
TCTTGGCTCTGGCATCGATGAAGAACGCAGCGAAATGCGATAAGTAATGTGAATTGCAGAATTCAGTGAATCA
TCGAATCTTTGAACGCACATTGCGCCCGCCGGCACTCCGGCGGGCATGCCTGTCCGAGCGTCATTTCAACCCT
CAGGACCCCCTTTCGGGGGGGACCTGGTGCTGGGGATCAGCGGCCTCCGGGCCCCTGTCCCCCAAATTGAGTG
GCGGTCGCGCCGCAGCCTCCCCTGCGTAGTAGCACACCTCGCACCGGAGAGCGGCTCGGCCACGCCGTGAAAC
CCCCAATTTTTTAAGGTTGACCTCGGATCAGGTAGGAATACCCGCTGAACTTAAGCATATCAAA

>Leaf mustard-2 25-17
TGACATACCTATCGTTCCCTCGGCGGGCTCAGCGCGCGGTGCCTCCGGGCTCCGGGCGTCCGCCGGGGACAAC
CAAACTCTGATTTTATTGTGAATCTCTGAGGGGCGAAAGCCCGACAACAAAATGAATCAAAACTTTCAACAAC
GGATCTCTTGGCTCTGGCATCGATGAAGAACGCAGCGAAATGCGATAAGTAATGTGAATTGCAGAATTCAGTG
AATCATCGAATCTTTGAACGCACATTGCGCCCGCCGGCACTCCGGCGGGCATGCCTGTCCGAGCGTCATTTCA
ACCCTCAGGACCCCCTTTCGGGGGGGACCTGGTGCTGGGGATCAGCGGCCTCCGGGCCCCTGTCCCCCAAATT
GAGTGGCGGTCGCGCCGCAGCCTCCCCTGCGTAGTAGCACACCTCGCACCGGAGAGCGGCTCGGCCACGCCGT
GAAACCCCCAATTTTTTAAGGTTGACCTCGGATCAGGTAGGAATACCCGCTGAACTTAAGCA
>Leaf mustard-2 28-3
TGACATACCTATCGTTCCCTCGGCGGGCTCAGCGCGCGGTGCCTCCGGGCTCCGGGCGTCCGCCGGGGACAAC
CAAACTCTGATTTTATTGTGAATCTCTGAGGGGCGAAAGCCCGACAACAAAATGAATCAAAACTTTCAACAAC
GGATCTCTTGGCTCTGGCATCGATGAAGAACGCAGCGAAATGCGATAAGTAATGTGAATTGCAGAATTCAGTG
AATCATCGAATCTTTGAACGCACATTGCGCCCGCCGGCACTCCGGCGGGCATGCCTGTCCGAGCGTCATTTCA
ACCCTCAGGACCCCCTTTCGGGGGGGACCTGGTGCTGGGGATCAGCGGCCTCCGGGCCCCTGTCCCCCAAATT
GAGTGGCGGTCGCGCCGCAGCCTCCCCTGCGTAGTAGCACACCTCGCACCGGAGAGCGGCTCGGCCACGCCGT
GAAACCCCCAATTTTTTAAGGTTGACCTCGGATCAGGTAGGAATACCCGCTGAACTTAAGCATAT
>Leaf mustard-2 28-4
TGACATACCTATCGTTCCCTCGGCGGGCTCAGCGCGCGGTGCCTCCGGGCTCCGGGCGTCCGCCGGGGACAAC
CAAACTCTGATTTTATTGTGAATCTCTGAGGGGCGAAAGCCCGACAACAAAATGAATCAAAACTTTCAACAAC
GGATCTCTTGGCTCTGGCATCGATGAAGAACGCAGCGAAATGCGATAAGTAATGTGAATTGCAGAATTCAGTG
AATCATCGAATCTTTGAACGCACATTGCGCCCGCCGGCACTCCGGCGGGCATGCCTGTCCGAGCGTCATTTCA
ACCCTCAGGACCCCCTTTCGGGGGGGACCTGGTGCTGGGGATCAGCGGCCTCCGGGCCCCTGTCCCCCAAATT
GAGTGGCGGTCGCGCCGCAGCCTCCCCTGCGTAGTAGCACACCTCGCACCGGAGAGCGGCTCGGCCACGCCGT
GAAACCCCCAATTTTTTAAGGTTGACCTCGGATCAGGTAGGAATACCCGCTGAACTTAAGCA
>Napa cabbage 25-1
CATACCTATCGTTCCCTCGGCGGGCTCAGCGCGCGGTGCCTCCGGGCTCCGGGCGTCCGCCGGGGACAACCAA
ACTCTGATTTTATTGTGAATCTCTGAGGGGCGAAAGCCCGACAACAAAATGAATCAAAACTTTCAACAACGGA
TCTCTTGGCTCTGGCATCGATGAAGAACGCAGCGAAATGCGATAAGTAATGTGAATTGCAGAATTCAGTGAAT
CATCGAATCTTTGAACGCACATTGCGCCCGCCGGCACTCCGGCGGGCATGCCTGTCCGAGCGTCATTTCAACC
CTCAGGACCCCCTTTCGGGGGGGACCTGGTGCTGGGGATCAGCGGCCTCCGGGCCCCTGTCCCCCAAATTGAG
TGGCGGTCGCGCCGCAGCCTCCCCTGCGTAGTAGCACACCTCGCACCGGAGAGCGGCTCGGCCACGCCGTGAA
ACCCCCAATTTTTTAAGGTTGACCTCGGATCAGGTAGGAATACCCGCTGAACTTAAGCATATCAA
>Napa cabbage 25-3
TTGTGAACATACCTATCGTTCCCTCGGCGGGCTCAGCGCGCGGTGCCTCCGGGCTCCGGGCGTCCGCCGGGGA
CAACCAAACTCTGATTTTATTGTGAATCTCTGAGGGGCGAAAGCCCGACAACAAAATGAATCAAAACTTTCAA
CAACGGATCTCTTGGCTCTGGCATCGATGAAGAACGCAGCGAAATGCGATAAGTAATGTGAATTGCAGAATTC
AGTGAATCATCGAATCTTTGAACGCACATTGCGCCCGCCGGCACTCCGGCGGGCATGCCTGTCCGAGCGTCAT
TTCAACCCTCAGGACCCCCTTTCGGGGGGGACCTGGTGCTGGGGATCAGCGGCCTCCGGGCCCCTGTCCCCCA
AATTGAGTGGCGGTCGCGCCGCAGCCTCCCCTGCGTAGTAGCACACCTCGCACCGGAGAGCGGCTCGGCCACG
CCGTGAAACCCCCAATTTTTTAAGGTTGACCTCGGATCAGGTAGGAATACCCGCTGAACTTAAGCA
>Napa cabbage 26-1
TTGTGAACATACCTATCGTTCCCTCGGCGGGCTCAGCGCGCGGTGCCTCCGGGCTCCGGGCGTCCGCCGGGGA
CAACCAAACTCTGATTTTATTGTGAATCTCTGAGGGGCGAAAGCCCGACAACAAAATGAATCAAAACTTTCAA
CAACGGATCTCTTGGCTCTGGCATCGATGAAGAACGCAGCGAAATGCGATAAGTAATGTGAATTGCAGAATTC
AGTGAATCATCGAATCTTTGAACGCACATTGCGCCCGCCGGCACTCCGGCGGGCATGCCTGTCCGAGCGTCAT
TTCAACCCTCAGGACCCCCTTTCGGGGGGGACCTGGTGCTGGGGATCAGCGGCCTCCGGGCCCCTGTCCCCCA
AATTGAGTGGCGGTCGCGCCGCAGCCTCCCCTGCGTAGTAGCACACCTCGCACCGGAGAGCGGCTCGGCCACG
CCGTGAAACCCCCAATTTTTTAAGGTTGACCTCGGATCAGGTAGGAATACCCGCTGAACTTAAGCAT
>Napa cabbage 26-2
TTGTGAACATACCTATCGTTCCCTCGGCGGGCTCAGCGCGCGGTGCCTCCGGGCTCCGGGCGTCCGCCGGGGA
CAACCAAACTCTGATTTTATTGTGAATCTCTGAGGGGCGAAAGCCCGACAACAAAATGAATCAAAACTTTCAA
CAACGGATCTCTTGGCTCTGGCATCGATGAAGAACGCAGCGAAATGCGATAAGTAATGTGAATTGCAGAATTC
AGTGAATCATCGAATCTTTGAACGCACATTGCGCCCGCCGGCACTCCGGCGGGCATGCCTGTCCGAGCGTCAT
TTCAACCCTCAGGACCCCCTTTCGGGGGGGACCTGGTGCTGGGGATCAGCGGCCTCCGGGCCCCTGTCCCCCA
AATTGAGTGGCGGTCGCGCCGCAGCCTCCCCTGCGTAGTAGCACACCTCGCACCGGAGAGCGGCTCGGCCACG
CCGTGAAACCCCCAATTTTTTAAGGTTGACCTCGGATCAGGTAGGAATACCCGCTGAACTTA
>Napa cabbage 27-1

TTGTGAACATACCTATCGTTCCCTCGGCGGGCTCAGCGCGCGGTGCCTCCGGGCTCCGGGCGTCCGCCGGGGA
CAACCAAACTCTGATTTTATTGTGAATCTCTGAGGGGCGAAAGCCCGACAACAAAATGAATCAAAACTTTCAA
CAACGGATCTCTTGGCTCTGGCATCGATGAAGAACGCAGCGAAATGCGATAAGTAATGTGAATTGCAGAATTC
AGTGAATCATCGAATCTTTGAACGCACATTGCGCCCGCCGGCACTCCGGCGGGCATGCCTGTCCGAGCGTCAT
TTCAACCCTCAGGACCCCCTTTCGGGGGGGACCTGGTGCTGGGGATCAGCGGCCTCCGGGCCCCTGTCCCCCA
AATTGAGTGGCGGTCGCGCCGCAGCCTCCCCTGCGTAGTAGCACACCTCGCACCGGAGAGCGGCTCGGCCACG
CCGTGAAACCCCCAATTTTTTAAGGTTGACCTCGGATCAGGTAGGAATACCCGCTGAACTTA
>Napa cabbage 27-2
TTGTGAACATACCTATCGTTCCCTCGGCGGGCTCAGCGCGCGGTGCCTCCGGGCTCCGGGCGTCCGCCGGGGA
CAACCAAACTCTGATTTTATTGTGAATCTCTGAGGGGCGAAAGCCCGACAACAAAATGAATCAAAACTTTCAA
CAACGGATCTCTTGGCTCTGGCATCGATGAAGAACGCAGCGAAATGCGATAAGTAATGTGAATTGCAGAATTC
AGTGAATCATCGAATCTTTGAACGCACATTGCGCCCGCCGGCACTCCGGCGGGCATGCCTGTCCGAGCGTCAT
TTCAACCCTCAGGACCCCCTTTCGGGGGGGACCTGGTGCTGGGGATCAGCGGCCTCCGGGCCCCTGTCCCCCA
AATTGAGTGGCGGTCGCGCCGCAGCCTCCCCTGCGTAGTAGCACACCTCGCACCGGAGAGCGGCTCGGCCACG
CCGTGAAACCCCCAATTTTTTAAGGTTGACCTCGGATCAGGTAGGAATACCCGCTGAACTTAAGCATAT
>Napa cabbage 27-3
TTGTGAACATACCTATCGTTCCCTCGGCGGGCTCAGCGCGCGGTGCCTCCGGGCTCCGGGCGTCCGCCGGGGA
CAACCAAACTCTGATTTTATTGTGAATCTCTGAGGGGCGAAAGCCCGACAACAAAATGAATCAAAACTTTCAA
CAACGGATCTCTTGGCTCTGGCATCGATGAAGAACGCAGCGAAATGCGATAAGTAATGTGAATTGCAGAATTC
AGTGAATCATCGAATCTTTGAACGCACATTGCGCCCGCCGGCACTCCGGCGGGCATGCCTGTCCGAGCGTCAT
TTCAACCCTCAGGACCCCCTTTCGGGGGGGACCTGGTGCTGGGGATCAGCGGCCTCCGGGCCCCTGTCCCCCA
AATTGAGTGGCGGTCGCGCCGCAGCCTCCCCTGCGTAGTAGCACACCTCGCACCGGAGAGCGGCTCGGCCACG
CCGTGAAACCCCCAATTTTTTAAGGTTGACCTCGGATCAGGTAGGAATACCCGCTGAACTTA
>Napa cabbage 29-1
AACATACCTATCGTTCCCTCGGCGGGCTCAGCGCGCGGTGCCTCCGGGCTCCGGGCGTCCGCCGGGGACAACC
AAACTCTGATTTTATTGTGAATCTCTGAGGGGCGAAAGCCCGACAACAAAATGAATCAAAACTTTCAACAACG
GATCTCTTGGCTCTGGCATCGATGAAGAACGCAGCGAAATGCGATAAGTAATGTGAATTGCAGAATTCAGTGA
ATCATCGAATCTTTGAACGCACATTGCGCCCGCCGGCACTCCGGCGGGCATGCCTGTCCGAGCGTCATTTCAA
CCCTCAGGACCCCCTTTCGGGGGGGACCTGGTGCTGGGGATCAGCGGCCTCCGGGCCCCTGTCCCCCAAATTG
AGTGGCGGTCGCGCCGCAGCCTCCCCTGCGTAGTAGCACACCTCGCACCGGAGAGCGGCTCGGCCACGCCGTG
AAACCCCCAATTTTTTAAGGTTGACCTCGGATCAGGGAGGAATACCCGCTGA
>Napa cabbage 29-2
TTGTGAACATACCTATCGTTCCCTCGGCGGGCTCAGCGCGCGGTGCCTCCGGGCTCCGGGCGTCCGCCGGGGA
CAACCAAACTCTGATTTTATTGTGAATCTCTGAGGGGCGAAAGCCCGACAACAAAATGAATCAAAACTTTCAA
CAACGGATCTCTTGGCTCTGGCATCGATGAAGAACGCAGCGAAATGCGATAAGTAATGTGAATTGCAGAATTC
AGTGAATCATCGAATCTTTGAACGCACATTGCGCCCGCCGGCACTCCGGCGGGCATGCCTGTCCGAGCGTCAT
TTCAACCCTCAGGACCCCCTTTCGGGGGGGACCTGGTGCTGGGGATCAGCGGCCTCCGGGCCCCTGTCCCCCA
AATTGAGTGGCGGTCGCGCCGCAGCCTCCCCTGCGTAGTAGCACACCTCGCACCGGAGAGCGGCTCGGCCACG
CCGTGAAACCCCCAATTTTTTAAGGTTGACCTCGGATCAGGTAGGAATACCCGCTGAACTTAAGCA
>Napa cabbage 29-3
TGTGAACATACCTATCGTTCCCTCGGCGGGCTCAGCGCGCGGTGCCTCCGGGCTCCGGGCGTCCGCCGGGGAC
AACCAAACTCTGATTTTATTGTGAATCTCTGAGGGGCGAAAGCCCGACAACAAAATGAATCAAAACTTTCAAC
AACGGATCTCTTGGCTCTGGCATCGATGAAGAACGCAGCGAAATGCGATAAGTAATGTGAATTGCAGAATTCA
GTGAATCATCGAATCTTTGAACGCACATTGCGCCCGCCGGCACTCCGGCGGGCATGCCTGTCCGAGCGTCATT
TCAACCCTCAGGACCCCCTTTCGGGGGGGACCTGGTGCTGGGGATCAGCGGCCTCCGGGCCCCTGTCCCCCAA
ATTGAGTGGCGGTCGCGCCGCAGCCTCCCCTGCGTAGTAGCACACCTCGCACCGGAGAGCGGCTCGGCCACGC
CGTGAAACCCCCAATTTTTTAAGGTTGACCTCGGATCAGGTAGGAATACCCGCTGAACTT
>Napa cabbage 29-4
TTGTGAACATACCTATCGTTCCCTCGGCGGGCTCAGCGCGCGGTGCCTCCGGGCTCCGGGCGTCCGCCGGGGA
CAACCAAACTCTGATTTTATTGTGAATCTCTGAGGGGCGAAAGCCCGACAACAAAATGAATCAAAACTTTCAA
CAACGGATCTCTTGGCTCTGGCATCGATGAAGAACGCAGCGAAATGCGATAAGTAATGTGAATTGCAGAATTC
AGTGAATCATCGAATCTTTGAACGCACATTGCGCCCGCCGGCACTCCGGCGGGCATGCCTGTCCGAGCGTCAT
TTCAACCCTCAGGACCCCCTTTCGGGGGGGACCTGGTGCTGGGGATCAGCGGCCTCCGGGCCCCTGTCCCCCA
AATTGAGTGGCGGTCGCGCCGCAGCCTCCCCTGCGTAGTAGCACACCTCGCACCGGAGAGCGGCTCGGCCACG
CCGTGAAACCCCCAATTTTTTAAGGTTGACCTCGGATCAGGTAGGAATACCCGCTGAAC
>Napa cabbage 29-5
TTGTGAACATACCTATCGTTCCCTCGGCGGGCTCAGCGCGCGGTGCCTCCGGGCTCCGGGCGTCCGCCGGGGA
CAACCAAACTCTGATTTTATTGTGAATCTCTGAGGGGCGAAAGCCCGACAACAAAATGAATCAAAACTTTCAA

CAACGGATCTCTTGGCTCTGGCATCGATGAAGAACGCAGCGAAATGCGATAAGTAATGTGAATTGCAGAATTC
AGTGAATCATCGAATCTTTGAACGCACATTGCGCCCGCCGGCACTCCGGCGGGCATGCCTGTCCGAGCGTCAT
TTCAACCCTCAGGACCCCCTTTCGGGGGGGACCTGGTGCTGGGGATCAGCGGCCTCCGGGCCCCTGTCCCCCA
AATTGAGTGGCGGTCGCGCCGCAGCCTCCCCTGCGTAGTAGCACACCTCGCACCGGAGAGCGGCTCGGCCACG
CCGAGAAACCCCAAATTTTTTAACGTCGACCCCGGATCAGGCACGAAT
>Napa cabbage 12.2-1
TACCTATCGTTCCCTCGGCGGGCTCAGCGCGCGGTGCCTCCGGGCTCCGGGCGTCCGCCGGGGACAACCAAAC
TCTGATTTTATTGTGAATCTCTGAGGGGCGAAAGCCCGACAACAAAATGAATCAAAACTTTCAACAACGGATC
TCTTGGCTCTGGCATCGATGAAGAACGCAGCGAAATGCGATAAGTAATGTGAATTGCAGAATTCAGTGAATCA
TCGAATCTTTGAACGCACATTGCGCCCGCCGGCACTCCGGCGGGCATGCCTGTCCGAGCGTCATTTCAACCCT
CAGGACCCCCTTTCGGGGGGGACCTGGTGCTGGGGATCAGCGGCCTCCGGGCCCCTGTCCCCCAAATTGAGTG
GCGGTCGCGCCGCAGCCTCCCCTGCGTAGTAGCACACCTCGCACCGGAGAGCGGCTCGGCCACGCCGTGAAAC
CCCCAATTTTTTAAGGTTGACCTCGGATCAGGTAGGAATACCCGC
>Radish-2 26-4
TGTGAACATACCTATCGTTCCCTCGGCGGGCTCAGCGCGCGGTGCCTCCGGGCTCCGGGCGTCCGCCGGGGAC
AACCAAACTCTGATTTTATTGTGAATCTCTGAGGGGCGAAAGCCCGACAACAAAATGAATCAAAACTTTCAAC
AACGGATCTCTTGGCTCTGGCATCGATGAAGAACGCAGCGAAATGCGATAAGTAATGTGAATTGCAGAATTCA
GTGAATCATCGAATCTTTGAACGCACATTGCGCCCGCCGGCACTCCGGCGGGCATGCCTGTCCGAGCGTCATT
TCAACCCTCAGGACCCCCTTTCGGGGGGGACCTGGTGCTGGGGATCAGCGGCCTCCGGGCCCCTGTCCCCCAA
ATTGAGTGGCGGTCGCGCCGCAGCCTCCCCTGCGTAGTAGCACACCTCGCACCGGAGAGCGGCTCGGCCACGC
CGTGAAACCCCCAATTTTTTAAGGTTGACCTCGGATCAGGTAGGAATACCCGCTGAACTTAA
>Radish-2 27-3
TTGTGAACATACCTATCGTTCCCTCGGCGGGCTCAGCGCGCGGTGCCTCCGGGCTCCGGGCGTCCGCCGGGGA
CAACCAAACTCTGATTTTATTGTGAATCTCTGAGGGGCGAAAGCCCGACAACAAAATGAATCAAAACTTTCAA
CAACGGATCTCTTGGCTCTGGCATCGATGAAGAACGCAGCGAAATGCGATAAGTAATGTGAATTGCAGAATTC
AGTGAATCATCGAATCTTTGAACGCACATTGCGCCCGCCGGCACTCCGGCGGGCATGCCTGTCCGAGCGTCAT
TTCAACCCTCAGGACCCCCTTTCGGGGGGGACCTGGTGCTGGGGATCAGCGGCCTCCGGGCCCCTGTCCCCCA
AATTGAGTGGCGGTCGCGCCGCAGCCTCCCCTGCGTAGTAGCACACCTCGCACCGGAGAGCGGCTCGGCCACG
CCGTGAAACCCCCAATTTTTTAAGGTTGACCTCGGATCAGGTAGGAATACCCGCTGAACTTAAGCATATCAA
>Radish-2 27-4
TGTGACATACCTATCGTTCCCTCGGCGGGCTCAGCGCGCGGTGCCTCCGGGCTCCGGGCGTCCGCCGGGGACA
ACCAAACTCTGATTTTATTGTGAATCTCTGAGGGGCGAAAGCCCGACAACAAAATGAATCAAAACTTTCAACA
ACGGATCTCTTGGCTCTGGCATCGATGAAGAACGCAGCGAAATGCGATAAGTAATGTGAATTGCAGAATTCAG
TGAATCATCGAATCTTTGAACGCACATTGCGCCCGCCGGCACTCCGGCGGGCATGCCTGTCCGAGCGTCATTT
CAACCCTCAGGACCCCCTTTCGGGGGGGACCTGGTGCTGGGGATCAGCGGCCTCCGGGCCCCTGTCCCCCAAA
TTGAGTGGCGGTCGCGCCGCAGCCTCCCCTGCGTAGTAGCACACCTCGCACCGGAGAGCGGCTCGGCCACGCC
GTGAAACCCCCAATTTTTTAAGGTTGACCTCGGATCAGGTAGGAATACCCGCTGAACTTAAGC
>Radish-2 27-7
TTGTGACATACCTATCGTTCCCTCGGCGGGCTCAGCGCGCGGTGCCTCCGGGCTCCGGGCGTCCGCCGGGGAC
AACCAAACTCTGATTTTATTGTGAATCTCTGAGGGGCGAAAGCCCGACAACAAAATGAATCAAAACTTTCAAC
AACGGATCTCTTGGCTCTGGCATCGATGAAGAACGCAGCGAAATGCGATAAGTAATGTGAATTGCAGAATTCA
GTGAATCATCGAATCTTTGAACGCACATTGCGCCCGCCGGCACTCCGGCGGGCATGCCTGTCCGAGCGTCATT
TCAACCCTCAGGACCCCCTTTCGGGGGGGACCTGGTGCTGGGGATCAGCGGCCTCCGGGCCCCTGTCCCCCAA
ATTGAGTGGCGGTCGCGCCGCAGCCTCCCCTGCGTAGTAGCACACCTCGCACCGGAGAGCGGCTCGGCCACGC
CGTGAAACCCCCAATTTTTTAAGGTTGACCTCGGATCAGGTAGGAATACCCGCTGAACTTAAGCATATCAA
>Radish-2 27-8
TGTGAACATACCTATCGTTCCCTCGGCGGGCTCAGCGCGCGGTGCCTCCGGGCTCCGGGCGTCCGCCGGGGAC
AACCAAACTCTGATTTTATTGTGAATCTCTGAGGGGCGAAAGCCCGACAACAAAATGAATCAAAACTTTCAAC
AACGGATCTCTTGGCTCTGGCATCGATGAAGAACGCAGCGAAATGCGATAAGTAATGTGAATTGCAGAATTCA
GTGAATCATCGAATCTTTGAACGCACATTGCGCCCGCCGGCACTCCGGCGGGCATGCCTGTCCGAGCGTCATT
TCAACCCTCAGGACCCCCTTTCGGGGGGGACCTGGTGCTGGGGATCAGCGGCCTCCGGGCCCCTGTCCCCCAA
ATTGAGTGGCGGTCGCGCCGCAGCCTCCCCTGCGTAGTAGCACACCTCGCACCGGAGAGCGGCTCGGCCACGC
CGTGAAACCCCCAATTTTTTAAGGTTGACCTCGGATCAGGTAGGAATACCCGCTGAACT
>Radish-2 29-4
TTGTGAACATACCTATCGTTCCCTCGGCGGGCTCAGCGCGCGGTGCCTCCGGGCTCCGGGCGTCCGCCGGGGA
CAACCAAACTCTGATTTTATTGTGAATCTCTGAGGGGCGAAAGCCCGACAACAAAATGAATCAAAACTTTCAA
CAACGGATCTCTTGGCTCTGGCATCGATGAAGAACGCAGCGAAATGCGATAAGTAATGTGAATTGCAGAATTC
AGTGAATCATCGAATCTTTGAACGCACATTGCGCCCGCCGGCACTCCGGCGGGCATGCCTGTCCGAGCGTCAT

TTCAACCCTCAGGACCCCCTTTCGGGGGGGACCTGGTGCTGGGGATCAGCGGCCTCCGGGCCCCTGTCCCCCA
AATTGAGTGGCGGTCGCGCCGCAGCCTCCCCTGCGTAGTAGCACACCTCGCACCGGAGAGCGGCTCGGCCACG
CCGTGAAACCCCCAATTTTTTAAGGTTGACCTCGGATCAGGTAGGAATACCCGCTGAACTTAAGCATATCAA
>Radish-2 29-5
TTGTGACATACCTATCGTTCCCTCGGCGGGCTCAGCGCGCGGTGCCTCCGGGCTCCGGGCGTCCGCCGGGGAC
AACCAAACTCTGATTTTATTGTGAATCTCTGAGGGGCGAAAGCCCGACAACAAAATGAATCAAAACTTTCAAC
AACGGATCTCTTGGCTCTGGCATCGATGAAGAACGCAGCGAAATGCGATAAGTAATGTGAATTGCAGAATTCA
GTGAATCATCGAATCTTTGAACGCACATTGCGCCCGCCGGCACTCCGGCGGGCATGCCTGTCCGAGCGTCATT
TCAACCCTCAGGACCCCCTTTCGGGGGGGACCTGGTGCTGGGGATCAGCGGCCTCCGGGCCCCTGTCCCCCAA
ATTGAGTGGCGGTCGCGCCGCAGCCTCCCCTGCGTAGTAGCACACCTCGCACCGGAGAGCGGCTCGGCCACGC
CGTGAAACCCCCAATTTTTTAAGGTTGACCTCGGATCAGGTAGGAATACCCGCTGAACTTAAGCATATCAA
>Radish-2 29-7
TGTGAACATACCTATCGTTCCCTCGGCGGGCTCAGCGCGCGGTGCCTCCGGGCTCCGGGCGTCCGCCGGGGAC
AACCAAACTCTGATTTTATTGTGAATCTCTGAGGGGCGAAAGCCCGACAACAAAATGAATCAAAACTTTCAAC
AACGGATCTCTTGGCTCTGGCATCGATGAAGAACGCAGCGAAATGCGATAAGTAATGTGAATTGCAGAATTCA
GTGAATCATCGAATCTTTGAACGCACATTGCGCCCGCCGGCACTCCGGCGGGCATGCCTGTCCGAGCGTCATT
TCAACCCTCAGGACCCCCTTTCGGGGGGGACCTGGTGCTGGGGATCAGCGGCCTCCGGGCCCCTGTCCCCCAA
ATTGAGTGGCGGTCGCGCCGCAGCCTCCCCTGCGTAGTAGCACACCTCGCACCGGAGAGCGGCTCGGCCACGC
CGTGAAACCCCCAATTTTTTAAGGTTGACCTCGGATCAGGTAGGAATACCCGCTGAACTTAAGCA
>Spinach 24-4
TTGTGAACATACCTATCGTTCCCTCGGCGGGCTCAGCGCGCGGTGCCTCCGGGCTCCGGGCGTCCGCCGGGGA
CAACCAAACTCTGATTTTATTGTGAATCTCTGAGGGGCGAAAGCCCGACAACAAAATGAATCAAAACTTTCAA
CAACGGATCTCTTGGCTCTGGCATCGATGAAGAACGCAGCGAAATGCGATAAGTAATGTGAATTGCAGAATTC
AGTGAATCATCGAATCTTTGAACGCACATTGCGCCCGCCGGCACTCCGGCGGGCATGCCTGTCCGAGCGTCAT
TTCAACCCTCAGGACCCCCTTTCGGGGGGGACCTGGTGCTGGGGATCAGCGGCCTCCGGGCCCCTGTCCCCCA
AATTGAGTGGCGGTCGCGCCGCAGCCTCCCCTGCGTAGTAGCACACCTCGCACCGGAGAGCGGCTCGGCCACG
CCGTGAAACCCCCAATTTTTTAAGGTTGACCTCGGATCAGGTAGGAATACCCGCTGAACTTAAGCA
>Spinach 24-6
TTGTGAACATACCTATCGTTCCCTCGGCGGGCTCAGCGCGCGGTGCCTCCGGGCTCCGGGCGTCCGCCGGGGA
CAACCAAACTCTGATTTTATTGTGAATCTCTGAGGGGCGAAAGCCCGACAACAAAATGAATCAAAACTTTCAA
CAACGGATCTCTTGGCTCTGGCATCGATGAAGAACGCAGCGAAATGCGATAAGTAATGTGAATTGCAGAATTC
AGTGAATCATCGAATCTTTGAACGCACATTGCGCCCGCCGGCACTCCGGCGGGCATGCCTGTCCGAGCGTCAT
TTCAACCCTCAGGACCCCCTTTCGGGGGGGACCTGGTGCTGGGGATCAGCGGCCTCCGGGCCCCTGTCCCCCA
AATTGAGTGGCGGTCGCGCCGCAGCCTCCCCTGCGTAGTAGCACACCTCGCACCGGAGAGCGGCTCGGCCACG
CCGTGAAACCCCCAATTTTTTAAGGTTGACCTCGGATCAGGTAGGAATACCCGCTGAACTTAAGCA
>Spinach 26-6
TTGTGAACATACCTATCGTTCCCTCGGCGGGCTCAGCGCGCGGTGCCTCCGGGCTCCGGGCGTCCGCCGGGGA
CAACCAAACTCTGATTTTATTGTGAATCTCTGAGGGGCGAAAGCCCGACAACAAAATGAATCAAAACTTTCAA
CAACGGATCTCTTGGCTCTGGCATCGATGAAGAACGCAGCGAAATGCGATAAGTAATGTGAATTGCAGAATTC
AGTGAATCATCGAATCTTTGAACGCACATTGCGCCCGCCGGCACTCCGGCGGGCATGCCTGTCCGAGCGTCAT
TTCAACCCTCAGGACCCCCTTTCGGGGGGGACCTGGTGCTGGGGATCAGCGGCCTCCGGGCCCCTGTCCCCCA
AATTGAGTGGCGGTCGCGCCGCAGCCTCCCCTGCGTAGTAGCACACCTCGCACCGGAGAGCGGCTCGGCCACG
CCGTGAAACCCCCAATTTTTTAAGGTTGACCTCGGATCAGGTAGGAATACCCGCTGAACTT
>Spinach 26-7
TTGTGAACATACCTATCGTTCCCTCGGCGGGCTCAGCGCGCGGTGCCTCCGGGCTCCGGGCGTCCGCCGGGGA
CAACCAAACTCTGATTTTATTGTGAATCTCTGAGGGGCGAAAGCCCGACAACAAAATGAATCAAAACTTTCAA
CAACGGATCTCTTGGCTCTGGCATCGATGAAGAACGCAGCGAAATGCGATAAGTAATGTGAATTGCAGAATTC
AGTGAATCATCGAATCTTTGAACGCACATTGCGCCCGCCGGCACTCCGGCGGGCATGCCTGTCCGAGCGTCAT
TTCAACCCTCAGGACCCCCTTTCGGGGGGGACCTGGTGCTGGGGATCAGCGGCCTCCGGGCCCCTGTCCCCCA
AATTGAGTGGCGGTCGCGCCGCAGCCTCCCCTGCGTAGTAGCACACCTCGCACCGGAGAGCGGCTCGGCCACG
CCGTGAAACCCCCAATTTTTTAAGGTTGACCTCGGATCACGTAGGAACACCCGCTGAA
>Spinach 26-8
TTGTGAACATACCTATCGTTCCCTCGGCGGGCTCAGCGCGCGGTGCCTCCGGGCTCCGGGCGTCCGCCGGGGA
CAACCAAACTCTGATTTTATTGTGAATCTCTGAGGGGCGAAAGCCCGACAACAAAATGAATCAAAACTTTCAA
CAACGGATCTCTTGGCTCTGGCATCGATGAAGAACGCAGCGAAATGCGATAAGTAATGTGAATTGCAGAATTC
AGTGAATCATCGAATCTTTGAACGCACATTGCGCCCGCCGGCACTCCGGCGGGCATGCCTGTCCGAGCGTCAT
TTCAACCCTCAGGACCCCCTTTCGGGGGGGACCTGGTGCTGGGGATCAGCGGCCTCCGGGCCCCTGTCCCCCA

AATTGAGTGGCGGTCGCGCCGCAGCCTCCCCTGCGTAGTAGCACACCTCGCACCGGAGAGCGGCTCGGCCACG
CCGTGAAACCCCCAATTTTTTAAGGTTGACCTCGGATCAGGT
>Spinach 27-1
AACATACCTATCGTTCCCTCGGCGGGCTCAGCGCGCGGTGCCTCCGGGCTCCGGGCGTCCGCCGGGGACAACC
AAACTCTGATTTTATTGTGAATCTCTGAGGGGCGAAAGCCCGACAACAAAATGAATCAAAACTTTCAACAACG
GATCTCTTGGCTCTGGCATCGATGAAGAACGCAGCGAAATGCGATAAGTAATGTGAATTGCAGAATTCAGTGA
ATCATCGAATCTTTGAACGCACATTGCGCCCGCCGGCACTCCGGCGGGCATGCCTGTCCGAGCGTCATTTCAA
CCCTCAGGACCCCCTTTCGGGGGGGACCTGGTGCTGGGGATCAGCGGCCTCCGGGCCCCTGTCCCCCAAATTG
AGTGGCGGTCGCGCCGCAGCCTCCCCTGCGTAGTAGCACACCTCGCACCGGAGAGCGGCTCGGCCACGCCGTG
AAACCCCCAATTTTTTAAGGTTGACCTCGGATCAGGTAGGAATACCCGCTGAACTTAAGCA
>Spinach 29-2
TTGTGAACATACCTATCGTTCCCTCGGCGGGCTCAGCGCGCGGTGCCTCCGGGCTCCGGGCGTCCGCCGGGGA
CAACCAAACTCTGATTTTATTGTGAATCTCTGAGGGGCGAAAGCCCGACAACAAAATGAATCAAAACTTTCAA
CAACGGATCTCTTGGCTCTGGCATCGATGAAGAACGCAGCGAAATGCGATAAGTAATGTGAATTGCAGAATTC
AGTGAATCATCGAATCTTTGAACGCACATTGCGCCCGCCGGCACTCCGGCGGGCATGCCTGTCCGAGCGTCAT
TTCAACCCTCAGGACCCCCTTTCGGGGGGGACCTGGTGCTGGGGATCAGCGGCCTCCGGGCCCCTGTCCCCCA
AATTGAGTGGCGGTCGCGCCGCAGCCTCCCCTGCGTAGTAGCACACCTCGCACCGGAGAGCGGCTCGGCCACG
CCGTGAAACCCCCAATTTTTTAAGGTTGACCTCGGATCAGGTAGGAATACCCGCTGAACTTA
>Spinach 29-3
AACATACCTATCGTTCCCTCGGCGGGCTCAGCGCGCGGTGCCTCCGGGCTCCGGGCGTCCGCCGGGGACAACC
AAACTCTGATTTTATTGTGAATCTCTGAGGGGCGAAAGCCCGACAACAAAATGAATCAAAACTTTCAACAACG
GATCTCTTGGCTCTGGCATCGATGAAGAACGCAGCGAAATGCGATAAGTAATGTGAATTGCAGAATTCAGTGA
ATCATCGAATCTTTGAACGCACATTGCGCCCGCCGGCACTCCGGCGGGCATGCCTGTCCGAGCGTCATTTCAA
CCCTCAGGACCCCCTTTCGGGGGGGACCTGGTGCTGGGGATCAGCGGCCTCCGGGCCCCTGTCCCCCAAATTG
AGTGGAGGTCGCGCCGCAGCCTCCCCTGCATGTATCACACCTCGTTCCGGATAGCGGCTCGGTCACCTCGATA
AACCCCCATTTTTTGATTTAGACCTCATGATTAAGCTCGAAAACCCCATTATTGCAACC
>Stem lettuce 24-4
TTGTGAACATACCTATCGTTCCCTCGGCGGGCTCAGCGCGCGGTGCCTCCGGGCTCCGGGCGTCCGCCGGGGA
CAACCAAACTCTGATTTTATTGTGAATCTCTGAGGGGCGAAAGCCCGACAACAAAATGAATCAAAACTTTCAA
CAACGGATCTCTTGGCTCTGGCATCGATGAAGAACGCAGCGAAATGCGATAAGTAATGTGAATTGCAGAATTC
AGTGAATCATCGAATCTTTGAACGCACATTGCGCCCGCCGGCACTCCGGCGGGCATGCCTGTCCGAGCGTCAT
TTCAACCCTCAGGACCCCCTTTCGGGGGGGACCTGGTGCTGGGGATCAGCGGCCTCCGGGCCCCTGTCCCCCA
AATTGAGTGGCGGTCGCGCCGCAGCCTCCCCTGCGTAGTAGCACACCTCGCACCGGAGAGCGGCTCGGCCACG
CCGTGAAACCCCCAATTTTTTAAGGTTGACCTCGGATCAGGTAGGAATACCCGCTGAACTTAAGCATAT
>Stem lettuce 12.2-2
TTGTGAACATACCTATCGTTCCCTCGGCGGGCTCAGCGCGCGGTGCCTCCGGGCTCCGGGCGTCCGCCGGGGA
CAACCAAACTCTGATTTTATTGTGAATCTCTGAGGGGCGAAAGCCCGACAACAAAATGAATCAAAACTTTCAA
CAACGGATCTCTTGGCTCTGGCATCGATGAAGAACGCAGCGAAATGCGATAAGTAATGTGAATTGCAGAATTC
AGTGAATCATCGAATCTTTGAACGCACATTGCGCCCGCCGGCACTCCGGCGGGCATGCCTGTCCGAGCGTCAT
TTCAACCCTCAGGACCCCCTTTCGGGGGGGACCTGGTGCTGGGGATCAGCGGCCTCCGGGCCCCTGTCCCCCA
AATTGAGTGGCGGTCGCGCCGCAGCCTCCCCTGCGTAGTAGCACACCTCGCACCGGAGAGCGGCTCGGCCACG
CCGTGAAACCCCCAATTTTTTAAGGTTGACCTCGGATCAGGTAGGAATACCCGCTGAACTTAAGCATAT

TEF1a sequences

>Bok choy 24-2
CGCTGCCGGTACTGGTGAGTTCGAGGCTGGTATCTCCAAGGATGGCCAGACTCGTGAGCACGCCCTGCTCGCC
TACACCCTCGGTGTGCGTCAGCTCATCGTCGCCATCAACAAGATGGACACCACCAAGTGGTCTGAGGCCCGTT
ACCAGGAGATTATCAAGGAGACCTCCAACTTCATCAAGAAGGTCGGCTACAACCCCAAGACCGTCGCCTTCGT
CCCCATCTCCGGTTTCCACGGTGACAACATGCTTGCCCCCACCACCAACGCCCCCTGGTACAAGGGTTGGGAG
CGTGAGATCAAGGGCAACAAGCAGACCGGCAAGACCCTCCTCGAGGCCATTGACGGTGTTGAGCCCCCCAAGC
GTCCCTCCGACAAGCCCCTCCGTCTTCCCCTTCAGGATGTCTACAAGATTGGTGGTATTGGCACGGTCCCTGT
CGGCCGTATCGAGACCGGTGTCCTCAAGCCCGGTATGGTCGTCACCTTCGCCCCTGCCAACGTCACCACTGAA
GTCAAGTCCGTCGAGATGCACCACGAGCAGCTCTCTGAGGGTCTTCCCGGTGACAATGT
>Bok choy 24-4
CGCTGCCGGTACTGGTGAGTTCGAGGCTGGTATCTCCAAGGATGGCCAGACTCGTGAGCACGCCCTGCTCGCC
TACACCCTCGGTGTGCGTCAGCTCATCGTCGCCATCAACAAGATGGACACCACCAAGTGGTCTGAGGCCCGTT
ACCAGGAGATTATCAAGGAGACCTCCAACTTCATCAAGAAGGTCGGCTACAACCCCAAGACCGTCGCCTTCGT
CCCCATCTCCGGTTTCCACGGTGACAACATGCTTGCCCCCACCACCAACGCCCCCTGGTACAAGGGTTGGGAG

CGTGAGATCAAGGGCAACAAGCAGACCGGCAAGACCCTCCTCGAGGCCATTGACGGTGTTGAGCCCCCCAAGC
GTCCCTCCGACAAGCCCCTCCGTCTTCCCCTTCAGGATGTCTACAAGATTGGTGGTATTGGCACGGTCCCTGT
CGGCCGTATCGAGACCGGTGTCCTCAAGCCCGGTATGGTCGTCACCTTCGCCCCTGCCAACGTCACCACTGAA
GTCAAGTCCGTCGAGATGCACCACGAGCAGCTCTCTGAGGGTCTTCCCGGTGACAA
>Radish-2 27-3
CGCTGCCGGTACTGGTGAGTTCGAGGCTGGTATCTCCAAGGATGGCCAGACTCGTGAGCACGCCCTGCTCGCC
TACACCCTCGGTGTGCGTCAGCTCATCGTCGCCATCAACAAGATGGACACCACCAAGTGGTCTGAGGCCCGTT
ACCAGGAGATTATCAAGGAGACCTCCAACTTCATCAAGAAGGTCGGCTACAACCCCAAGACCGTCGCCTTCGT
CCCCATCTCCGGTTTCCACGGTGACAACATGCTTGCCCCCACCACCAACGCCCCCTGGTACAAGGGTTGGGAG
CGTGAGATCAAGGGCAACAAGCAGACCGGCAAGACCCTCCTCGAGGCCATTGACGGTGTTGAGCCCCCCAAGC
GTCCCTCCGACAAGCCCCTCCGTCTTCCCCTTCAGGATGTCTACAAGATTGGTGGTATTGGCACGGTCCCTGT
CGGCCGTATCGAGACCGGTGTCCTCAAGCCCGGTATGGTCGTCACCTTCGCCCCTGCCAACGTCACCACTGAA
GTCAAGTCCGTCGAGATGCACCACGAGCAGCTCTCTGAGGGTCTTCCCGGTGACAATGT
>Radish-2 27-4
CGCTGCCGGTACTGGTGAGTTCGAGGCTGGTATCTCCAAGGATGGCCAGACTCGTGAGCACGCCCTGCTCGCC
TACACCCTCGGTGTGCGTCAGCTCATCGTCGCCATCAACAAGATGGACACCACCAAGTGGTCTGAGGCCCGTT
ACCAGGAGATTATCAAGGAGACCTCCAACTTCATCAAGAAGGTCGGCTACAACCCCAAGACCGTCGCCTTCGT
CCCCATCTCCGGTTTCCACGGTGACAACATGCTTGCCCCCACCACCAACGCCCCCTGGTACAAGGGTTGGGAG
CGTGAGATCAAGGGCAACAAGCAGACCGGCAAGACCCTCCTCGAGGCCATTGACGGTGTTGAGCCCCCCAAGC
GTCCCTCCGACAAGCCCCTCCGTCTTCCCCTTCAGGATGTCTACAAGATTGGTGGTATTGGCACGGTCCCTGT
CGGCCGTATCGAGACCGGTGTCCTCAAGCCCGGTATGGTCGTCACCTTCGCCCCTGCCAACGTCACCACTGAA
GTCAAGTCCGTCGAGATGCACCACGAGCAGCTCTCTGAGGGTCTTCCCGGTGACAATGT
>Radish-2 27-7
CGCTGCCGGTACTGGTGAGTTCGAGGCTGGTATCTCCAAGGATGGCCAGACTCGTGAGCACGCCCTGCTCGCC
TACACCCTCGGTGTGCGTCAGCTCATCGTCGCCATCAACAAGATGGACACCACCAAGTGGTCTGAGGCCCGTT
ACCAGGAGATTATCAAGGAGACCTCCAACTTCATCAAGAAGGTCGGCTACAACCCCAAGACCGTCGCCTTCGT
CCCCATCTCCGGTTTCCACGGTGACAACATGCTTGCCCCCACCACCAACGCCCCCTGGTACAAGGGTTGGGAG
CGTGAGATCAAGGGCAACAAGCAGACCGGCAAGACCCTCCTCGAGGCCATTGACGGTGTTGAGCCCCCCAAGC
GTCCCTCCGACAAGCCCCTCCGTCTTCCCCTTCAGGATGTCTACAAGATTGGTGGTATTGGCACGGTCCCTGT
CGGCCGTATCGAGACCGGTGTCCTCAAGCCCGGTATGGTCGTCACCTTCGCCCCTGCCAACGTCACCACTGAA
GTCAAGTCCGTCGAGATGCACCACGAGCAGCTCTCTGAGGGTCTTCCCGGTGACAATGT
>Radish-2 27-8
CGCTGCCGGTACTGGTGAGTTCGAGGCTGGTATCTCCAAGGATGGCCAGACTCGTGAGCACGCCCTGCTCGCC
TACACCCTCGGTGTGCGTCAGCTCATCGTCGCCATCAACAAGATGGACACCACCAAGTGGTCTGAGGCCCGTT
ACCAGGAGATTATCAAGGAGACCTCCAACTTCATCAAGAAGGTCGGCTACAACCCCAAGACCGTCGCCTTCGT
CCCCATCTCCGGTTTCCACGGTGACAACATGCTTGCCCCCACCACCAACGCCCCCTGGTACAAGGGTTGGGAG
CGTGAGATCAAGGGCAACAAGCAGACCGGCAAGACCCTCCTCGAGGCCATTGACGGTGTTGAGCCCCCCAAGC
GTCCCTCCGACAAGCCCCTCCGTCTTCCCCTTCAGGATGTCTACAAGATTGGTGGTATTGGCACGGTCCCTGT
CGGCCGTATCGAGACCGGTGTCCTCAAGCCCGGTATGGTCGTCACCTTCGCCCCTGCCAACGTCACCACTGAA
GTCAAGTCCGTCGAGATGCACCACGAGCAGCTCTCTGAGGGTCTTCCCGGTGACAC
>Radish-2 29-4
TATCGCTGCCGGTACTGGTGAGTTCGAGGCTGGTATCTCCAAGGATGGCCAGACTCGTGAGCACGCCCTGCTC
GCCTACACCCTCGGTGTGCGTCAGCTCATCGTCGCCATCAACAAGATGGACACCACCAAGTGGTCTGAGGCCC
GTTACCAGGAGATTATCAAGGAGACCTCCAACTTCATCAAGAAGGTCGGCTACAACCCCAAGACCGTCGCCTT
CGTCCCCATCTCCGGTTTCCACGGTGACAACATGCTTGCCCCCACCACCAACGCCCCCTGGTACAAGGGTTGG
GAGCGTGAGATCAAGGGCAACAAGCAGACCGGCAAGACCCTCCTCGAGGCCATTGACGGTGTTGAGCCCCCCA
AGCGTCCCTCCGACAAGCCCCTCCGTCTTCCCCTTCAGGATGTCTACAAGATTGGTGGTATTGGCACGGTCCC
TGTCGGCCGTATCGAGACCGGTGTCCTCAAGCCCGGTATGGTCGTCACCTTCGCCCCTGCCAACGTCACCACT
GAAGTCAAGTCCGTCGAGATGCACCACGAGCAGCTCTCTGAGGGTCTTCCCGGGAC
>Radish-2 29-5
CGCTGCCGGTACTGGTGAGTTCGAGGCTGGTATCTCCAAGGATGGCCAGACTCGTGAGCACGCCCTGCTCGCC
TACACCCTCGGTGTGCGTCAGCTCATCGTCGCCATCAACAAGATGGACACCACCAAGTGGTCTGAGGCCCGTT
ACCAGGAGATTATCAAGGAGACCTCCAACTTCATCAAGAAGGTCGGCTACAACCCCAAGACCGTCGCCTTCGT
CCCCATCTCCGGTTTCCACGGTGACAACATGCTTGCCCCCACCACCAACGCCCCCTGGTACAAGGGTTGGGAG
CGTGAGATCAAGGGCAACAAGCAGACCGGCAAGACCCTCCTCGAGGCCATTGACGGTGTTGAGCCCCCCAAGC
GTCCCTCCGACAAGCCCCTCCGTCTTCCCCTTCAGGATGTCTACAAGATTGGTGGTATTGGCACGGTCCCTGT
CGGCCGTATCGAGACCGGTGTCCTCAAGCCCGGTATGGTCGTCACCTTCGCCCCTGCCAACGTCACCACTGAA
GTCAAGTCCGTCGAGATGCACCACGAGCAGCTCTCTGAGGGTCTTCCCGGTGACAATGT

>Spinach 27-1
ATCGCTGCCGGTACTGGTGAGTTCGAGGCTGGTATCTCCAAGGATGGCCAGACTCGTGAGCACGCCCTGCTCG
CCTACACCCTCGGTGTGCGTCAGCTCATCGTCGCCATCAACAAGATGGACACCACCAAGTGGTCTGAGGCCCG
TTACCAGGAGATTATCAAGGAGACCTCCAACTTCATCAAGAAGGTCGGCTACAACCCCAAGACCGTCGCCTTC
GTCCCCATCTCCGGTTTCCACGGTGACAACATGCTTGCCCCCACCACCAACGCCCCCTGGGACAAGGGTTGGG
AGCGTGAGATCAAGGGCAACAAGCAGACCGGGAAGACCCTCCTCGAGGTCATTGACGGTGTTGAGCCCCCCAA
GCGTCCCTCCGACAAGCCCCTCCGTCTTCCCCTTCAGGATGTCTACAAGATTGGTGGTATTGGCACGGTCCCT
GCGGGCCCGTATCGAGACCGGTGTCCTCAAGCCCGGTATGGTCGTCACCTTCGCCCCTGCCAACGTCACCACT
GAAGTCAAGTCCGTCGAGATGCACCACGAGCAGCTCTCTGAGGGTCTTCCCGGTGAC
>Stem lettuce 24-20
CGCTGCCGGTACTGGTGAGTTCGAGGCTGGTATCTCCAAGGATGGCCAGACTCGTGAGCACGCCCTGCTCGCC
TACACCCTCGGTGTGCGTCAGCTCATCGTCGCCATCAACAAGATGGACACCACCAAGTGGTCTGAGGCCCGTT
ACCAGGAGATTATCAAGGAGACCTCCAACTTCATCAAGAAGGTCGGCTACAACCCCAAGACCGTCGCCTTCGT
CCCCATCTCCGGTTTCCACGGTGACAACATGCTTGCCCCCACCACCAACGCCCCCTGGTACAAGGGTTGGGAG
CGTGAGATCAAGGGCAACAAGCAGACCGGCAAGACCCTCCTCGAGGCCATTGACGGTGTTGAGCCCCCCAAGC
GTCCCTCCGACAAGCCCCTCCGTCTTCCCCTTCAGGATGTCTACAAGATTGGTGGTATTGGCACGGTCCCTGT
CGGCCGTATCGAGACCGGTGTCCTCAAGCCCGGTATGGTCGTCACCTTCGCCCCTGCCAACGTCACCACTGAA
GTCAAGTCCGTCGAGATGCACCACGAGCAGCTCTCTGAGGGTCTTCCCGGTGACAATGTCGGATTCAACGTC
>Stem lettuce 24-26
CGCTGCCGGTACTGGTGAGTTCGAGGCTGGTATCTCCAAGGATGGCCAGACTCGTGAGCACGCCCTGCTCGCC
TACACCCTCGGTGTGCGTCAGCTCATCGTCGCCATCAACAAGATGGACACCACCAAGTGGTCTGAGGCCCGTT
ACCAGGAGATTATCAAGGAGACCTCCAACTTCATCAAGAAGGTCGGCTACAACCCCAAGACCGTCGCCTTCGT
CCCCATCTCCGGTTTCCACGGTGACAACATGCTTGCCCCCACCACCAACGCCCCCTGGTACAAGGGTTGGGAG
CGTGAGATCAAGGGCAACAAGCAGACCGGCAAGACCCTCCTCGAGGCCATTGACGGTGTTGAGCCCCCCAAGC
GTCCCTCCGACAAGCCCCTCCGTCTTCCCCTTCAGGATGTCTACAAGATTGGTGGTATTGGCACGGTCCCTGT
CGGCCGTATCGAGACCGGTGTCCTCAAGCCCGGTATGGTCGTCACCTTCGCCCCTGCCAACGTCACCACTGAA
GTCAAGTCCGTCGAGATGCACCACGAGCAGCTCTCTGAGGGTCTTCCCGGTGACAACG
>Stem lettuce 12.2-2
CGCTGCCGGTACTGGTGAGTTCGAGGCTGGTATCTCCAAGGATGGCCAGACTCGTGAGCACGCCCTGCTCGCC
TACACCCTCGGTGTGCGTCAGCTCATCGTCGCCATCAACAAGATGGACACCACCAAGTGGTCTGAGGCCCGTT
ACCAGGAGATTATCAAGGAGACCTCCAACTTCATCAAGAAGGTCGGCTACAACCCCAAGACCGTCGCCTTCGT
CCCCATCTCCGGTTTCCACGGTGACAACATGCTTGCCCCCACCACCAACGCCCCCTGGTACAAGGGTTGGGAG
CGTGAGATCAAGGGCAACAAGCAGACCGGCAAGACCCTCCTCGAGGCCATTGACGGTGTTGAGCCCCCCAAGC
GTCCCTCCGACAAGCCCCTCCGTCTTCCCCTTCAGGATGTCTACAAGATTGGTGGTATTGGCACGGTCCCTGT
CGGCCGTATCGAGACCGGTGTCCTCAAGCCCGGTATGGTCGTCACCTTCGCCCCTGCCAACGTCACCACTGAA
GTCAAGTCCGTCGAGATGCACCACGAGCAGCTCTCTGAGGGTCTTCCCGGTGACAATGT

***Lecanicillium* sp.**

ITS sequences
>Radish-2 27-16
TACCTTACAGTTGCTTCGGCGGAGCCGCCCCGGCGCCCGGAACCCAGTTTCGCGGCCCGGACCAAGGCGCCCG
CCGGAGGCCACAAACTCTTCTGTTTTTACAGTATCTTCTGAGTGTGCCGCAAGGCAAAAACAAATGAATCAAA
ACTTTCAACAACGGATCTCTTGGTTCTGGCATCGATGAAGAACGCAGCGAAATGCGATAAGTAATGTGAATTG
CAGAATTCAGTGAATCATCGAATCTTTGAACGCACATTGCGCCCGCCAGAATTCTGGCGGGCATGCCTGTTCG
AGCGTCATTTCAACCCTCGGTCTCCCTCCGGGAGAGACCGGCGTTGGGGACCGGCACTTACCCCGCCGGCCCC
GAAATGAAGTGGCGGCCCGTCCGCGGCGACCTCTGCGTAGTAACTCCACTCGCACCGGGACCCGGGCGCGGCC
ACGCCGTAAAACCCCCAACTTCCGAATGTTGACCTCGAATCAGGTAGGAATACCCGCTGAACTTAAGCATATC
>Celery 27-2
TACCTTACAGTTGCTTCGGCGGAGCCGCCCCGGCGCCCGGAACCCAGTTTCGCGGCCCGGACCAAGGCGCCCG
CCGGAGGCCACAAACTCTTCTGTTTTTACAGTATCTTCTGAGTGTGCCGCAAGGCAAAAACAAATGAATCAAA
ACTTTCAACAACGGATCTCTTGGTTCTGGCATCGATGAAGAACGCAGCGAAATGCGATAAGTAATGTGAATTG
CAGAATTCAGTGAATCATCGAATCTTTGAACGCACATTGCGCCCGCCAGAATTCTGGCGGGCATGCCTGTTCG
AGCGTCATTTCAACCCTCGGTCTCCCTCCGGGAGAGACCGGCGTTGGGGACCGGCACTTACCCCGCCGGCCCC
GAAATGAAGTGGCGGCCCGTCCGCGGCGACCTCTGCGTAGTAACTCCACTCGCACCGGGACCCGGGCGCGGCC
ACGCCGTAAAACCCCCAACTTCCGAATGTTGACCTCGAATCAGGTAGGAATACCCGCTGAACTTAAGCATAT

TEF1a sequences
>Radish-2 27-16
CATCGCTGCCGGTACTGGTGAGTTCGAGGCTGGTATCTCCAAGGATGGCCAGACCCGTGAGCACGCTCTCCTC
GCCTACACCCTGGGTGTCAAGCAGATCATTGTCGCCATCAACAAGATGGACACCACCAAGTGGTCTGAGGAGC
GTTACCAGGAAATCATCAAGGAGACCTCCAACTTCATCAAGAAGGTCGGCTACAACCCCAAGAACGTTGCCTT
CGTCCCCATCTCTGGCTTCAACGGTGACAACATGCTGTCTCCCTCCACCAACTGCCCCTGGTACAAGGGTTGG
GAGCGTGAGGGCAAGAATGGCAAGGTCACTGGCAAGACTCTCCTTGAGGCCATTGACTCCATCGAGCCCCCCA
AGCGTCCCTCCGACAAGCCCCTCCGTCTTCCCCTCCAGGATGTCTACAAGATCGGTGGTATCGGAACGGTCCC
TGTCGGCCGTGTCGAGACTGGTGTGATCAAGCCCGGCATGGTTGTCACCTTCGCCCCCGCTGGTGTCACCACT
GAAGTCAAGTCCGTCGAGATGCACCACGAGCAGCTCCCCGAGGGTGTCCCCGGT
>Celery 27-2
TGCCGGTACTGGTGAGTTCGAGGCTGGTATCTCCAAGGATGGCCAGACCCGTGAGCACGCTCTCCTCGCCTAC
ACCCTGGGTGTCAAGCAGATCATTGTCGCCATCAACAAGATGGACACCACCAAGTGGTCTGAGGAGCGTTACC
AGGAAATCATCAAGGAGACCTCCAACTTCATCAAGAAGGTCGGCTACAACCCCAAGAACGTTGCCTTCGTCCC
CATCTCTGGCTTCAACGGTGACAACATGCTGTCTCCCTCCACCAACTGCCCCTGGTACAAGGGTTGGGAGCGT
GAGGGCAAGAATGGCAAGGTCACTGGCAAGACTCTCCTTGAGGCCATTGACTCCATCGAGCCCCCCAAGCGTC
CCTCCGACAAGCCCCTCCGTCTTCCCCTCCAGGATGTCTACAAGATCGGTGGTATCGGAACGGTCCCTGTCGG
CCGTGTCGAGACTGGTGTGATCAAGCCCGGCATGGTTGTCACCTTCGCCCCCGCTGGTGTCACCACTGAAGTC
AAGTCCGTCGAGATGCACCACGAGCAGCTCCCCGAGGGTGTCCCCGGTGAC

***Alternaria* sp.**

ITS sequences
>Radish curly 29-6-ITS
ACAGCCTTGCTGAATTATTCACCCTTGTCTTTTGCGTACTTCTTGTTTCCTTGGTGGGTTCGCCCACCACTAG
GACAAACATAAACCTTTTGTAATTGCAATCAGCGTCAGTAACAAATTAATAATTACAACTTTCAACAACGGAT
CTCTTGGTTCTGGCATCGATGAAGAACGCAGCGAAATGCGATAAGTAGTGTGAATTGCAGAATTCAGTGAATC
ATCGAATCTTTGAACGCACATTGCGCCCTTTGGTATTCCAAAGGGCATGCCTGTTCGAGCGTCATTTGTACCC
TCAAGCTTTGCTTGGTGTTGGGCGTCTTGTCTCTAGCTTTGCTGGAGACTCGCCTTAAAGTAATTGGCAGCCG
GCCTACTGGTTTCGGAGCGCAGCACAAGTCGCACTCTCTATCAGCAAAGGTCTAGCATCCATTAAGCCTTTTT
TTCAACTTTTGACCTCGGATCAGGTAGGGATACCCGCTGAACTTAAGCATAT
>Spinach 29-1-ITS
TCTCGGGGTTACAGCCTTGCTGAATTATTCACCCTTGTCTTTTGCGTACTTCTTGTTTCCTTGGTGGGTTCGC
CCACCACTAGGACAAACATAAACCTTTTGTAATTGCAATCAGCGTCAGTAACAAATTAATAATTACAACTTTC
AACAACGGATCTCTTGGTTCTGGCATCGATGAAGAACGCAGCGAAATGCGATAAGTAGTGTGAATTGCAGAAT
TCAGTGAATCATCGAATCTTTGAACGCACATTGCGCCCTTTGGTATTCCAAAGGGCATGCCTGTTCGAGCGTC
ATTTGTACCCTCAAGCTTTGCTTGGTGTTGGGCGTCTTGTCTCTAGCTTTGCTGGAGACTCGCCTTAAAGTAA
TTGGCAGCCGGCCTACTGGTTTCGGAGCGCAGCACAAGTCGCACTCTCTATCAGCAAAGGTCTAGCATCCATT
AAGCCTTTTTTTCAACTTTTGACCTCGGATCAGGTAGGGATACCCGCTGAACTTAAGCATAT

TEF1a sequences
>Radish-2 29-6
TGGTGAGTTCGAGGCTGGTATCTCCAAGGATGGCCAGACTCGTGAGCACGCTCTCCTCGCTTACACCCTCGGT
GTCAAGCAGCTCATCGTTGCCATCAACAAGATGGACACCACCAAGTGGTCCGAGGAGCGTTACCAGGAGATCA
TCAAGGAGACCTCCAACTTCATCAAGAAGGTCGGCTACAACCCCAAGCACGTTCCCTTCGTCCCCATCTCCGG
TTTCAACGGTGACAACATGATTGAGGCCTCATCCAACTGCCCCTGGTACAAGGGTTGGGAGAAGGAGACCAAG
GCCAAGGCCACTGGTAAGACCCTCCTCGAGGCCATCGACGCCATCGACCCTCCCAGCCGTCCCACCGACAAGC
CCCTCCGTCTTCCCCTCCAGGATGTTTACAAGATTGGTGGTATTGGCACGGTGCCCGTCGGTCGTGTCGAGAC
CGGTATCATCAAGGCCGGTATGGTCGTCACCTTCGCCCCCGCTGGTGTCACCACTGAAGTCAAGTCCGTCGAG
ATGCACCACGAGCAGCTCACCGAGGGTGT
>Spinach 29-1
TGAGTTCGAGGCTGGTATCTCCAAGGATGGCCAGACTCGTGAGCACGCTCTCCTCGCTTACACCCTCGGTGTC
AAGCAGCTCATCGTTGCCATCAACAAGATGGACACCACCAAGTGGTCCGAGGAGCGTTACCAGGAGATCATCA
AGGAGACCTCCAACTTCATCAAGAAGGTCGGCTACAACCCCAAGCACGTTCCCTTCGTCCCCATCTCCGGTTT
CAACGGTGACAACATGATTGAGGCCTCATCCAACTGCCCCTGGTACAAGGGTTGGGAGAAGGAGACCAAGGCC
AAGGCCACTGGTAAGACCCTCCTCGAGGCCATCGACGCCATCGACCCTCCCAGCCGTCCCACCGACAAGCCCC

TCCGTCTTCCCCTCCAGGATGTTTACAAGATTGGTGGTATTGGCACGGTGCCCGTCGGTCGTGTCGAGACCGG
TATCATCAAGGCCGGTATGGTCGTCACCTTCGCCCCCGCTGGTGTCACCACTGAAGTCAAGTCCGTCGAGATG
CACCACGAGCAGCTCACCGAGGGTGTCCCCGGTG
