# Supplementary material for: Common but Nonpersistent Acquisitions of Plant Viruses by Plant-Associated Fungi
Source: Viruses. 2022 Oct 17;14(10):2279. doi: 10.3390/v14102279 (PMC9611831; doi:10.3390/v14102279)
Supplement: Supplementary file 1 [file viruses-14-02279-s001.zip › viruses-1937532-supplementary/Supplementary Table S1.pdf]

Supplementary Table 1. A list of primers used in this study.

Virus primer	Sequence	Product size（bp）
BnRV1-F	ATCCTAGTTACTGTGCTAAT	738
BnRV1-R	CGTAACGTACCCTTAAAATC	
PRSV-F	AACCCGAGCTTGTGGAGCCC	780
PRSV-R	TGATCAATTCGACAGGGATG	
WMV-F	ATCAATGAAGCAAGTTAGTC	738
WMV-R	ACAAAGTACGGTAGTGTAGT	
ZYMV-F	TCGTATTGAGCAGGATGAAG	738
ZYMV-R	AAGGTTGCTGCTAACTCACC	
KoMV-F	ACCTCCAAAAACTCCTCAAC	760
KoMV-R	ACTTAATGAGTTTAATGCGG	
QRV3-F	ATTGAAGGTTTATGTTGTTT	738
QRV3-R	GACTCTTTATCTTTTCTTCT	
QRV4-F	ACTCACAGCAAACTTTGTGT	738
QRV4-R	TTGTCAGACTTATCTATGTC	
TuMV-F	ATTTGCTCATGTCACTGGTG	738
TuMV-R	TTCTTGGTAATGTAACGGTG	
BBWV-2-F	GATTTCAGGATCAATCAAGA	738
BBWV-2-R	CCCAGAAGAATTGTGAGATC	
QRV1-F	ATCTTGAACAGTACGCTATC	742
QRV1-R	CTTGTGTTACAGGCTTCATT	
Ixeridium-F	ATTCCAGTCGTAGCTGGCAC	738
Ixeridium-R	TGGACTCCATTGCCTGTTGA	
QRV2-F	AAGTTCAGTCTTGAGAATTG	738
QRV2-R	TGCCCCTACTATAAGCAAGT	
CMV-F	ATGCGCCACGCGGAACGTTT	738
CMV-R	CATCCACGCTTTCTTATCAT	
BrYV-F	ATCGAGCTTCGCTTGTTTAT	738
BrYV-R	AGTGGTCGAACAGATTTCCG	
TuYV-aRNA-F	ATCTGGGCTCATCGGGGAGG	738
TuYV-aRNA-R	GTTGAGGATAGTATAGATAG	

Virus primer (nested PCR)	Sequence	Product size（bp）
BnRV1-CF	GTGTCAGTCCGTAATCCCTAA	445
BnRV1-CR	TTGCTTGAGAAGAAGTCCC	
ZYMV-CF	GTTTGCGATCACCGTTCT	438
ZYMV-CR	CAATCCCGTCGTCGTCTC	
QRV3-CF	TCTAAGCAATCTGTCATC	413
QRV3-CR	CTCCCATCATAAGAAGTA	
QRV4-1338-CR	ATTCAGGTCATTAGGTGTTCC	431
QRV4-908-CF	TATTTATGGTTGCGGTCC	
TuMV-CF	GTAATGTAACGGTGAAGCAGG	413
TuMV-CR	TGTTCGCTGTATGGAGTTGA	
BBWV-2-CF	CGCAGCTACGTGTATTGTC	434
BBWV-2-CR	CGAAATAAATCATGTCCCTC	
QRV1-CF	TTCTGACTTATCCGATGTTTCT	440
QRV1-CR	ATCAGTTTGACCTCCGTTG	
CMV-CF	TGGGCTTTAGAGGATTATGTG	420
CMV-CR	TGAGTTCGGGTGGGCATA	
BrYV-CF	AGTGTTCCTCCTCGGGTTG	432
BrYV-CR	CGAAAGCGATGAAGACACCAA	
TuYV-aRNA-CF	ACCAACCGCCAACTCGTC	447
TuYV-aRNA-CR	GCAGGGAGCGCAACATAA	

Fungal primer	Sequence
ITS1-F	TCCGTAGGTGAACCTGCGG
ITS4-R	TCCTCCGCTTATTGATATGC
EF1-1018F	GAYTTCATCAAGAACATGAT
EF1-1620R	GACGTTGAADCCRACRTTGTC
